# Sensorimotor content of multi-unit activity recorded in the paramedian lobule of the cerebellum using carbon fiber microelectrode arrays

**DOI:** 10.3389/fnins.2024.1232653

**Published:** 2024-02-29

**Authors:** Esma Cetinkaya, Eric J. Lang, Mesut Sahin

**Affiliations:** ^1^Biomedical Engineering Department, New Jersey Institute of Technology, Newark, NJ, United States; ^2^Department of Neuroscience and Physiology, NYU Neuroscience Institute, New York University Grossman School of Medicine, New York, NY, United States

**Keywords:** cerebellar electrophysiology, carbon fiber electrodes, reaching behavior, local field potentials, chronic neural recording

## Abstract

The cerebellum takes in a great deal of sensory information from the periphery and descending signals from the cerebral cortices. It has been debated whether the paramedian lobule (PML) in the rat and its paravermal regions that project to the interpositus nucleus (IPN) are primarily involved in motor execution or motor planning. Studies that have relied on single spike recordings in behaving animals have led to conflicting conclusions regarding this issue. In this study, we tried a different approach and investigated the correlation of field potentials and multi-unit signals recorded with multi-electrode arrays from the PML cortex along with the forelimb electromyography (EMG) signals in rats during behavior. Linear regression was performed to predict the EMG signal envelopes using the PML activity for various time shifts (±25, ±50, ±100, and ± 400 ms) between the two signals to determine a causal relation. The highest correlations (~0.5 on average) between the neural and EMG envelopes were observed for zero and small (±25 ms) time shifts and decreased with larger time shifts in both directions, suggesting that paravermal PML is involved both in processing of sensory signals and motor execution in the context of forelimb reaching behavior. EMG envelopes were predicted with higher success rates when neural signals from multiple phases of the behavior were utilized for regression. The forelimb extension phase was the most difficult to predict while the releasing of the bar phase prediction was the most successful. The high frequency (>300 Hz) components of the neural signal, reflecting multi-unit activity, had a higher contribution to the EMG prediction than did the lower frequency components, corresponding to local field potentials. The results of this study suggest that the paravermal PML in the rat cerebellum is primarily involved in the execution of forelimb movements rather than the planning aspect and that the PML is more active at the initiation and termination of the behavior, rather than the progression.

## 1 Introduction

For over a century, it has been known that the cerebellum plays a vital role in the generation of well-coordinated movements and that its injury causes disturbances of voluntary movement (Holmes, 1917). While its specific contributions to motor control continue to be debated (Ebner et al., 2011), hypotheses focus on its role in motor learning (Gilbert and Thach, 1977), timing (Welsh et al., 1995), and its instantiation of internal models for prediction and error correction (Popa and Ebner, 2019).

Many single-unit recordings in behaving animals have shown cerebellar neurons modulate their firing rates during movement and this modulation can be well-correlated to various movement parameters (Fuchs et al., 1993; Goossens et al., 2004; Servais et al., 2004; Schonewille et al., 2006; Bryant et al., 2009). Results indicate that PML is a good target for studying correlations between reaching behavior and cerebellar activity based on its connectivity patterns (Apps and Hawkes, 2009). Moreover, simple spike (SS) activity from the rat PML modulates during the trained reaching task (Heck et al., 2007) and monkey interpositus nucleus, a target of the PML, shows increased discharge during reaching and grasping (Kan et al., 1993). However, most prior studies were limited to single-unit recordings, which do not provide direct insight into the relationships across the cells. To look at population activity, one could do simultaneous multi-unit recording or alternatively look at field potentials during movements. This information may be a key to understanding the role of cerebellar output, since timing and synchronization are population activities, and both theoretical and experimental studies suggest that they are vital in shaping cerebellar output (Maex and Schutter, 1998; Shin and Schutter, 2006; Schutter and Steuber, 2009; Blenkinsop and Lang, 2011; Lang and Blenkinsop, 2011; Person and Raman, 2012; Hong et al., 2016; Tang et al., 2016).

To obtain multi-unit recordings in behaving animals, we built novel microelectrode arrays using extremely thin (7 μm diameter) carbon fibers to record multi-unit activity (MUA) and local field potentials (LFPs) from the rat cerebellar cortex with minimal disturbance to the neural tissue (Cetinkaya et al., 2018; Cetinkaya, 2022), and used these arrays to record from the paravermal regions of the PML during a reaching task. We were able to predict EMG activity from the PML activity, which supports the hypothesis that paravermal PML is involved in processing sensorimotor information. Furthermore, we conducted a detailed analysis of the reaching behavior considering its phases and sensorimotor content, and frequency band contributions of the paravermal PML during behavior.

## 2 Methodology

### 2.1 Electrode fabrication

#### 2.1.1 Carbon fiber microelectrode arrays (CFMEAs)

**Figure 1:**
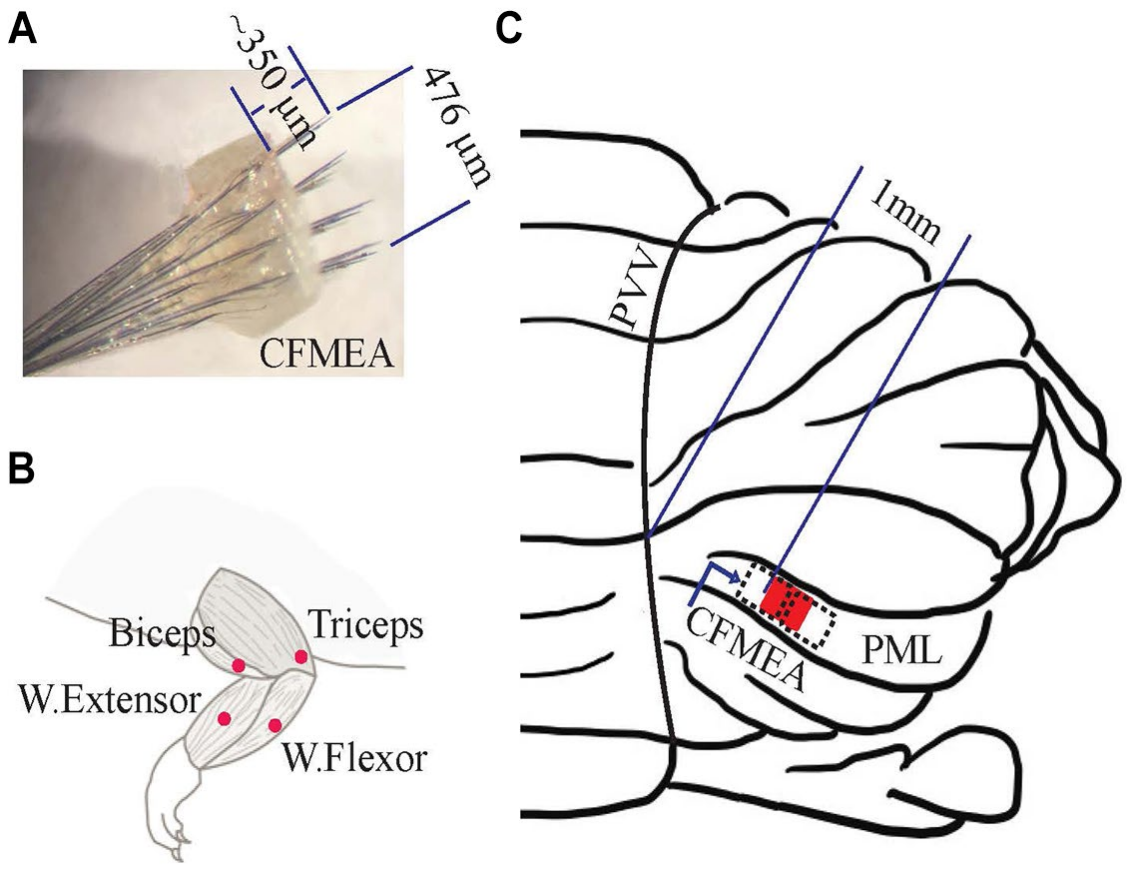
Implantation of the neural electrode array on the cerebellar cortex and EMG electrodes on the forelimb muscles. **(A)** Neural Electrode (CFMEA: carbon fiber microelectrode array). One of the fabricated carbon fiber microelectrode arrays with 32 channels (4 × 8). It covers an area of 476 μm in the mediolateral direction and 408 μm in the rostrocaudal direction. **(B)** EMG Electrode Implantation Area. The forelimb muscles [wrist flexor (ventral), wrist extensor (dorsal), biceps (elbow flexor), and triceps (elbow extensor)] that received subcutaneous EMG electrodes. **(C)** Electrode (CFMEA) Position on the PML of Rat Cerebellum (red filled box and black dash boxes). Its medial edge is ~1 mm from the paravermal vein (PVV) in the paramedian lobule (PML) for 6 animals (red box), 0.75 mm, and 1.25 mm for other animals (dash boxes).

The arrays were constructed using carbon fibers (C005711/2, 7 μm diam., Goodfellow Cambridge Ltd., England) that were coated with 2 μm (±50%) parylene-C at a foundry (Specialty Coating Systems, IN) at room temperature. Single carbon fibers were cut to 1.5–3 cm under a microscope. About 250 μm of the parylene-C coating was removed from one end of the filament using a soldering iron heated to the parylene-C melting temperature (290°C). The uncoated end of each filament was then connected to a pin on a micro-connector (A79022-001, Omnetics Connector Corporation, MN) using conductive silver epoxy (EpoTek, MA). Once 32 filaments were attached to the micro-connector, the epoxy was cured by placing the entire assembly in an oven at 200°C for 10 min. Next, a nine-by-ten matrix was cut from a nylon woven mesh sheet with a thread diameter of 37 μm and 31 μm hole size (U-CMN-31, Component Supply, TN). Single carbon fibers were inserted through the holes in the mesh to form a 4 × 8 array (136 μm x 68 μm pitch) with a total footprint of 408 μm (rostrocaudal) x 476 μm (mediolateral). The fibers were then fixed in place in the mesh with medical epoxy (Figure 1A) (OJ2116 EpoTek, MA). The ends of the carbon fibers protruding on the other side of the mesh were cut to a length of ~400 μm using a computer-controlled laser system (450 nm blue laser, 4 W, Wercan Comp., China). This carbon fiber length was chosen to be a little longer than the PC layer depth (~250 μm in the rat) to allow for post-implantation tissue growth under the electrode substrate that had been observed in prior experiments. Reference and ground electrodes were constructed from one-centimeter-long Teflon-coated multi-strand stainless steel wires (25.4 μm, 793,200, A-M Systems, WA) that were deinsulated for a few mm from their ends. Once all fibers and grounds were connected, the entire micro-connector assembly was covered with medical epoxy to insulate and protect the connections. *In vitro* impedances of individual carbon fibers were measured [2.07 ± 0.45 MΩ, mean ± SD (standard deviation)], *n* = 320 carbon fibers in each array with an analog impedance meter (BAK Electronics, IMP-2, FL). If >20% of channels had impedances out of the expected impedance range (1–5 MΩ), the array was not used for implantation.

#### 2.1.2 EMG electrodes

EMG wire electrodes were constructed from 5 cm long Teflon-coated multi-strand stainless steel wire pairs that were connected to an Omnetics connector (A79022-001) and coated with clear medical epoxy (EpoTek, OJ2116, MA) for hermetic sealing. The tips of the wires were deinsulated for ~2 mm and staggered by 3 mm to enable differential recording, and medical epoxy was applied to the very end forming a small bead to provide an anchor for suturing to the muscle belly epimysially and to prevent the sharp wire ends from causing irritation or itching of the overlying skin.

### 2.2 Animals

All procedures were approved by the Institutional Animal Care and Use Committee (IACUC), Rutgers University, Newark, NJ. Eight male Long Evans rats (~175 g initial weight) were placed on a food restriction diet and then trained on a reach-to-pull task. Once they learned the task, they underwent surgery to implant EMG electrodes to the forelimb muscles and carbon fiber microelectrode arrays to the cerebellum. Following the post-surgical recovery period, neural, EMG, and behavioral data were collected while animals performed the behavioral task. Food restriction of animals began 3 days before training to boost the animals’ attentiveness to training and appetite for sugar pellets. Animals were kept on the food restricted diet throughout the experiment, except for a period surrounding the implantation surgery (from 2 days prior to 4 days after surgery), during which time they were on an *ad libitum* diet. During food restriction, animals were initially fed three food pellets per day (LabDiet, 5,001 Rodent Diet, ~15 g, IN) in addition to receiving ~13.5 g of sugar pellet rewards (Bio-Serv, F0021, Dustless Precision Pellets, NJ) during each training/recording session. The animals’ weights were monitored during training and recording, and food pellet feeding was adjusted, if necessary, to keep their weights within ±20% of their initial weight. Animals generally weighed between 180–250 g during the time of data collection.

#### 2.2.1 Behavioral task

**Figure 2:**
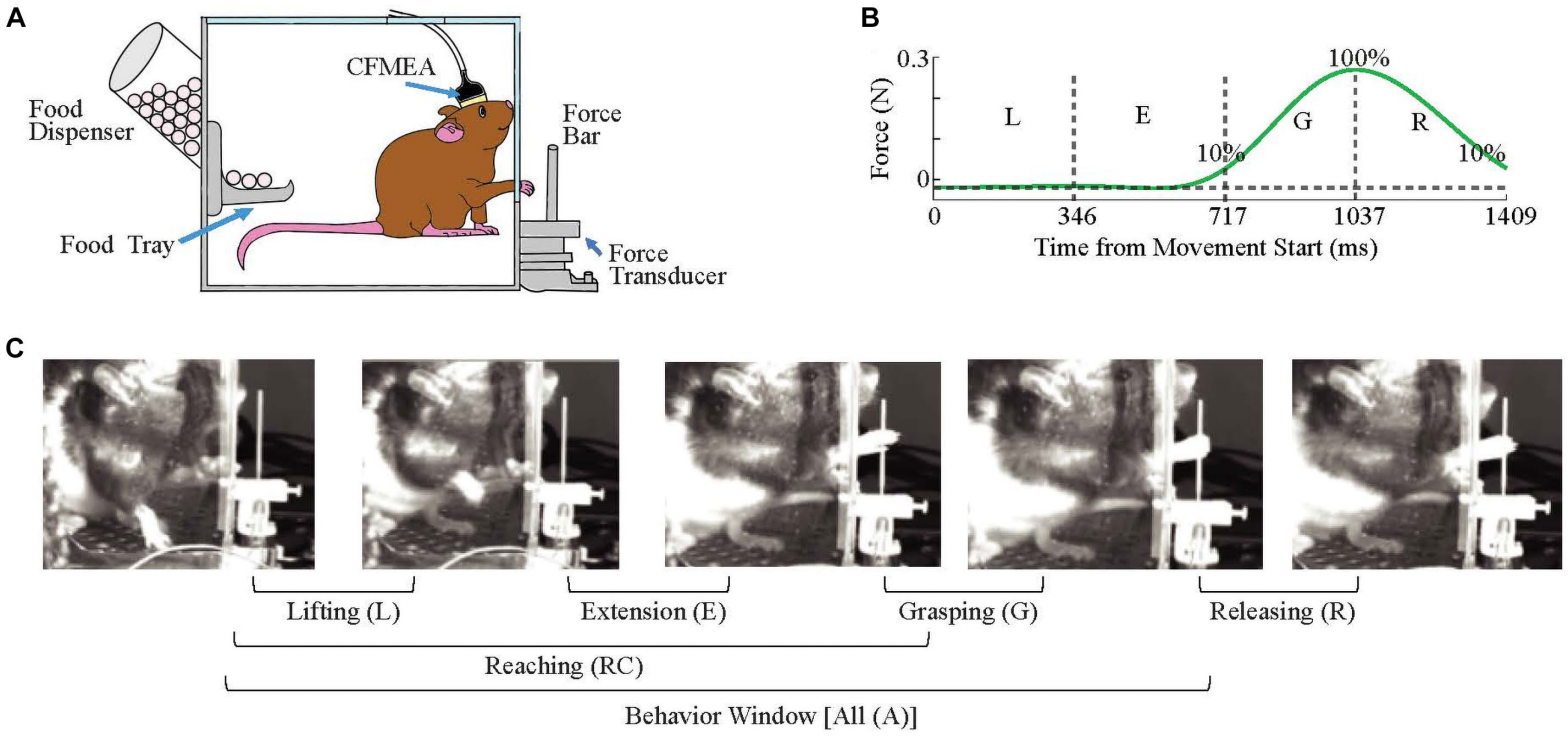
Behavioral Task. **(A)** Recording Setup. **(B)** A Representative Force Profile. The time windows corresponding to all four phases of the behavior were marked. Approximate time points for lifting (L) and extension (E) phases are generated from video images, and for grasping (G) and releasing (R) from the force data (n = 39 trials, eight animals). The start of grasping and end of releasing phases are the timepoints at which it reaches 10% of maximum force amplitude. Vertical dash lines separate phases while horizontal dash line indicates baseline force. **(C)** Four Stages of the Reaching Movement.

The training apparatus consisted of a clear plexiglass box (width x height x depth, 30 cm x 26 cm x 22 cm) with a food dispenser and tray mounted on one wall and a vertically-oriented 20 mm wide slit in the opposing wall that ran from the level of the transducer platform (~ 3 cm from the chamber floor) to the top of the chamber (Figure 2A). A vertical bar (38 mm height, 1 mm radius) (called ‘force bar’ throughout the paper) that was connected to a force transducer (Nano 17, ATI Industrial Automation) was fixed outside the box at a distance of 13 mm from the box wall and center-aligned with the slit. The animal’s movements during the task were recorded with a video camera positioned on the side of the box, between the force bar and the slit. The behavioral task was a reach-to-pull task in which the animal was trained to extend a forelimb through the slit to reach and grab the force bar and pull it with a threshold force of ~0.25 N.

#### 2.2.2 Shaping procedure

In the first session, animals were acclimated to the training/recording box by placing them in the box for 1–2 h and putting sugar pellets in the tray. In the following sessions, sugar pellets were initially held with a tweezer behind the force bar to induce the animal to reach through the slit and touch the bar. The animal was rewarded for any reaching attempt with sugar pellets being placed in the tray (sessions 2–3). Once the animal’s paw was regularly contacting the force bar, sugar pellets were no longer held behind the bar by the experimenter. Instead, the force threshold was set to a minimum level so that the computerized system released three sugar pellets to the food tray upon any contact of the bar and played a tone (883 Hz and 1.3 s duration) to signal the delivery of a reward. The threshold force was then incrementally increased to ~0.25 N over the next 1–2 sessions. The animal’s preferred limb was noted during these initial training sessions, and if the animal started using the other limb during the recording sessions, the slit was partially blocked to force the animal to use the same limb as in the initial training sessions. Forelimb reaches were self-initiated, subject only to a 10-s dead-time following each reach for which a reward was given. In each session, an animal typically initiated ~75 trials. Training sessions typically lasted ~1–2 h and ended when the animal stopped making reaches for ~5 min. Training sessions were halted, and surgery was performed to implant the electrodes when the animal was making >50 successful trials per session for 2–3 sessions in a row. The training time was about 2 weeks (~5–6 sessions).

### 2.3 Surgery

Anesthesia was induced with 5% isoflurane (v/v) and maintained with 2.0–2.5% isoflurane in 100% oxygen throughout the surgery. The animal’s head was fixed in a stereotaxic frame, and the fur in the surgical areas was shaved. Petrolatum ophthalmic vet ointment (Dechra, TX) was applied to the eyes to prevent dehydration. Blood oxygenation level and heart rate were monitored with a pulse oximeter attached to the hind paw. Body temperature was regulated at 36.5°C using a heating pad underneath the animal and a rectal temperature probe. Salix (0.5 mL/kg, IM) was given to avoid brain edema and bulging of the cerebellum during surgery. Local analgesic [Marcaine, (Bupivacaine HCl), 0.5 mL/kg, 2.5 mg/mL] was injected into the neck and head muscles around the incision areas as pre-emptive anesthesia. After making an incision to expose the skull from the bregma to the lambda and removing the periosteum completely, five to six holes were drilled into the skull using a fine drill bit. Metal screws (0–80 × 1/16, 1.6 mm, PlasticsOne, VA) were coated with a small amount of cyanoacrylate (Gluture, WPI, FL) and screwed into the holes. The skull was prepared by applying 3% hydrogen peroxide, followed by rinsing with normal saline and drying with gauze. The skull top and the screw heads were covered with dental acrylic (Ortho-Jet BCA, Lang Dental Manufacturing, IL) mixed with Gluture before both neural and EMG Omnetics connectors were fixed atop the skull using dental acrylic.

#### 2.3.1 EMG electrode implantation

Following fixation of the connectors to the skull, bipolar EMG electrodes were implanted to four muscles (wrist flexors, wrist extensors, biceps, and triceps, Figure 1B) of the preferred forelimb in order to record the activity of muscles that were recruited during the task. Four small skin incisions were made to expose the forelimb muscles. Before implanting each EMG electrode, the target muscle was electrically stimulated using a bipolar electrode (two tungsten wires separated by 1–2 mm). Stimuli were delivered using a current pulse generator (Model 2,200, A-M Systems, WA), and twitching of the targeted muscle was confirmed visually (wrist flexion/extension or elbow flexion/extension). The EMG electrode wire pairs were then tunneled subcutaneously from the Omnetics connector to their respective muscles with the help of a stainless-steel tube. Wrist flexor and extensor EMG electrodes were implanted near the midpoint of the forearm on the ventral and dorsal sides, respectively, while biceps and triceps EMG electrodes were implanted close to the elbow joint. The EMG wires were sutured with 8–0 non-absorbable sutures to the muscles epimysially and stimulated through the implanted EMG electrodes to reaffirm the muscle function. Finally, the skin incisions were closed with 6–0 absorbable sutures.

#### 2.3.2 Neural electrode (CFMEA) implantation and monitoring

Six animals were implanted with a single electrode array to the PML. In two other animals, two arrays were implanted next to each other on the PML. To implant the arrays, the muscles connecting to the occipital bone were detached on one side (ipsilateral to the preferred forelimb during training), from the suture with the interparietal bone down to about 2–3 mm from the foramen magnum, and from the midline to ~5 mm lateral. A cranial hole (2 × 2 mm) was opened over the PML in the posterior cerebellum using ophthalmic rongeurs. The dura was resected mediolaterally along the midline of PML. A 4×8 carbon fiber array was implanted by slowly inserting the fibers into the PML cortex to a depth of 250–300 μm. The medial edge of the array was located ~1.0 mm from the paravermal vein (total 6 arrays) when a single array was implanted, and ~ 0.75 and ~ 1.25 mm when two arrays were implanted next to each other mediolaterally (total 4 arrays) (Figure 1C). The carbon fibers extending from the Omnetics connector to the implanted array were covered with dental acrylic, except for the distal ~5 mm where the wires entered the mesh to form the array. Then, the top of the array was covered with connective tissue excised from the neck muscles to secure it in position. The reference and ground wires were fixed to the skull bone using dental acrylic. Finally, the skin incisions were closed with absorbable sutures. The skin edges around the connectors were tightly sealed to the dental acrylic base that held the connectors. *In vivo,* carbon fiber electrode impedances were measured and stored with Trellis software (Ripple Inc., UT) (2.46 ± 3.15 MΩ, n = 320 fibers) regularly throughout the recording period. They were concurrently monitored with the electrophysiological signals to judge if electrodes were failing, which would require an early termination of the study in a particular animal. None of the animals used in this paper required early termination.

### 2.4 Data collection

Neural and EMG recordings were collected at a 30 kHz sampling rate using a multi-channel head-stage (Grapevine front end nano2, Ripple Inc., UT) and amplifier (Scout processor, Ripple Inc., UT). The force signal was acquired at a 30 kHz sampling rate via a data acquisition card (PCI 6071, National Instruments, TX) controlled by a custom-written script in Matlab (Mathworks Inc., MA). Forearm movements were captured (initially at 100 frames/s but later at 30 frames/s due to storage issues) using a video camera (1080p HD Webcam C018, TeckNet, UK). All data recordings were synchronized using a TTL signal generated by the force transducer when the detected force increased above the threshold during a successful attempt. The study period (from the date of implantation to the last recording date) was 62.9 ± 19 days for all animals (n = 8 animals). A session was conducted every 6.4 ± 5.5 days, for a total of 82 sessions across all animals. The recording sessions were evenly spread over about a period of two months to demonstrate the reproducibility of results while tissue encapsulation around the neural electrodes was taking place. Each recording session consisted of ~65 trials, where each successful trial contained a single pull on the force bar. All data signals were recorded for 4 s, centered around the TTL trigger signal from the force transducer. Animals generally performed 3–4 trials/min, and one recording session took around 1–1.5 h, including a brief period of isoflurane anesthesia pre- and post-recording (to connect and detach the amplifier headstage, respectively), and impedance measurement.

### 2.5 Data analysis

All data processing and analysis of neural and EMG signals were performed in Matlab. Video analysis was done in Igor Pro (Version 8.0, WaveMetrics, OR).

#### 2.5.1 The phases of the reach-to-pull behavior

**Figure 3:**
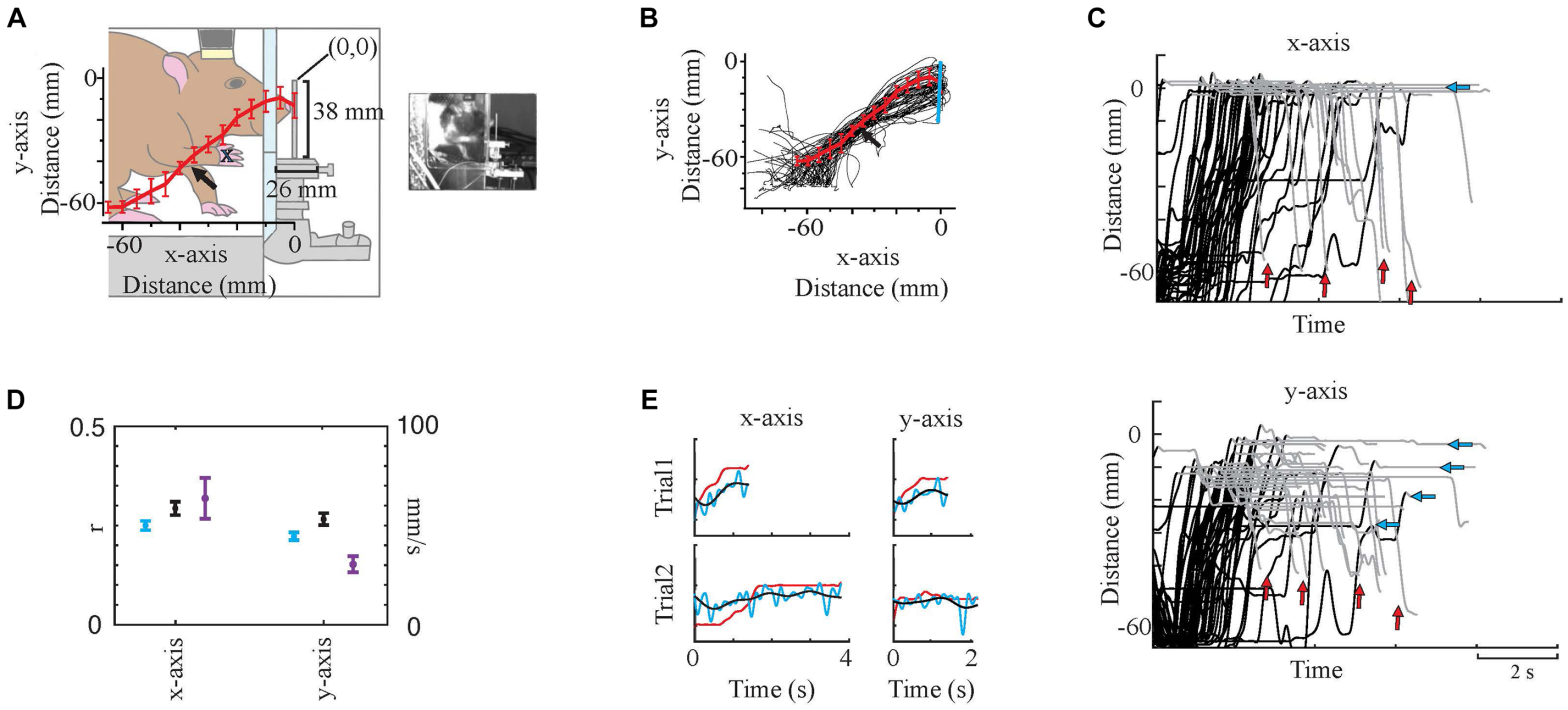
Video Analysis. **(A)** Simplified paw traces, the average trace (red line), and standard errors (bin size: 5 mm, red bars). The arrow shows a shift in the trajectory in some trials around the elbow level. The animal and surroundings were traced from the screenshot on the right. **(B)** shows all individual traces (black) with mean (red line plot) and SD (red bars). The blue vertical line represents the force bar. **(C)** Paw Tracking. Dorsoventral displacement (y-axis) vs. time, and rostrocaudal displacement (x-axis) vs. time. Black portions of the traces are L and E phases, while gray portions are G and R. Note: 0 is the tip of the force bar in both x- and y-axes that is marked on **(A)**. Blue arrows mark the end position(s) in the y-axis. Red arrows show the varying holding times. **(D)** Prediction correlations [Blue plots are original prediction results while black plots are filtered prediction trace results as in **(E)**] and speed (purple traces) in x- and y-axes. Dots are mean and whisker plots are standard error. **(E)** Sample x- and y-trace predictions on two trials. Red traces are measured trajectories while blue (original) and black (low-pass filtered) are predicted trajectories. These samples were from a test set of 11 trials. r values for original and filtered prediction traces with their measured x-traces were respectively, 0.32 and 0.42. For the y-traces r values were 0.24 and 0.36, respectively. y-axis of the plots is the displacement (mm).

For analysis purposes, a behavior window was defined as starting at the time at which the animal lifting its forearm from the floor, continuing through the reaching, and grabbing of the force bar, and ending when the animal breaks contact with the force bar. This behavior window was subdivided into four phases. The first phase is the lifting phase, and it starts coincident with the behavior window, when the animal removes its forelimb from the floor (Figure 2C, 1st image) and ends when the paw reaches the height of the elbow (Figure 2C, 2nd image). The extension phase follows immediately from the end of the lifting phase and ends when the animal touches the force bar (Figure 2C, 3rd image). The decision to divide the reaching motion into an early lifting and later extension phase was based on video records from individual trials that showed that in some trials, there is a slight bend in the trajectory when the paw reaches close to elbow level (Figure 3A, arrow). The end of the extension phase marks the beginning of the grasping phase, which ends when the bar is fully grasped (Figure 2C, 4th image), which, in turn, marks the beginning of the releasing phase. Finally, the releasing phase ends when the animal’s paw lets go of the bar, which is also the end of the behavior window.

##### 2.5.1.1 Determining the timestamps of phases on every trial

The beginning and end of the behavior window, and four phases of the reaching movement within this window, were determined using a combination of the video records and force profiles. Frame-by-frame analysis of the video records for all trials was not physically feasible, so the average durations of first two phases were estimated from frame-by-frame analysis of a sample of trials. The lifting and extension phases lasted for 346 ± 301 ms and 371 ± 447 ms on average, respectively [n = 39 trials, sampled from eight animals, Figure 2B. High SD can be due to across animal differences or the variations in speed (see Figure 3D)]. To simplify the analysis, both phases were approximated as 350 ms long. This approximation was validated for a limited subset of recording sessions in which frame-by-frame analysis was used to exactly mark the movement phases (see section ‘Video-Based Sessions’).

The force profiles were then used to mark the specific start and end times of the grasping and releasing phases (Figure 2B). The grasping phase start was set to when the force amplitude rises above 10% of the maximum and the end was defined as the time of the force peak. The releasing phase starts at the force peak and ends when the force amplitude drops below 10% of the maximum. The grasping start time was then used as a fiduciary point to set both the start of the extension phase by subtracting 350 ms from it and the start of the lifting phase (and overall behavior window) but subtracting another 350 ms. Analyses were then performed for each of the four movement phases, the entire behavior window (‘All’), and the combined first three phases (lifting, extension, and grasping), which we refer to as the reaching phase.

#### 2.5.2 Video analysis

Video images were used for four purposes in addition to helping define the movement phases described above.

1. *Eliminating Mistrials.* Images were used to check whether the behavior was performed according to the trained pattern in each trial. Each trial’s video recording was monitored, and if a trial showed deviations, like using two hands or a mis-triggering of the system by touching the force transducer platform rather than the bar, it was eliminated.
2. *Reach Trajectory (Displacement Tracing).* The paw trajectories in x- and y-axes during each trial were measured by analyzing the video file (mp4) using Igor Pro (Version 8.0, WaveMetrics, OR). The paw trajectories were manually traced in the side view video recordings (n = 54 trials from one session of one animal) by tracking the point of the stem of the second and third digits (Figure 3A blue cross indicates the traced position). Displacement was traced frame by frame (30 frames per second) in a 60 mm by 60 mm region of interest. The average number of frames traced per trial was 113. Tracing started 2–3 frames before the animal started lifting its paw and completed when the animal pulled back its paw to the elbow level after releasing the force bar. The force bar tip was assigned as zero on both x- and y-axes. Then the paw coordinates were converted from pixels to mm, and the traces were resampled (250 Hz) to get an equal number of samples in each trial to allow us to calculate the average and SD (bin size 5 mm) of points along the trajectories (Figures 3A,B). Lastly, they were 5 Hz low-pass filtered (4^th^-order Butterworth filter) to smoothen them.
3. *Wrist and Elbow Angles.* Knuckle, shoulder, elbow, and wrist trajectories were traced in time frame-by-frame, similar to reach trajectory tracing (n = 19, multiple sessions in three animals). Trajectories of different sessions/animals were aligned using the force bar location as a reference to account for slight day-to-day changes *in camera* position. The elbow and wrist angles were computed from these trajectories. The elbow angle was measured between the line that connects the elbow to the wrist and the line that connects the elbow to the shoulder, while for the wrist angle, the lines were the one between the wrist and knuckle and the other one between the wrist and the elbow.
4. *Video-Based Sessions.* Lifting and the extension phases` initiation and termination time points were marked for three sessions from three different animals (n = 154 trials) based on the definitions in section 2.5.1. The EMG signal prediction was performed for a second time using these trial-specific time marks and was compared to the original approach which was using average time windows for lifting and extension phases.

#### 2.5.3 Processing of neural signals and artifact elimination

##### 2.5.3.1 Differential signal calculation

To get rid of common-mode signals in the neural arrays, differential signals were calculated by pairing the 32 electrodes in an array to form 16 differential channels. Electrode pairing for differential channels was based on their positions in the array as the closest neighboring channels in the mediolateral direction (medial channel-lateral channel).

##### 2.5.3.2 Movement artifact removal

The neural signals further cleared of short duration (<50 ms) movement artifacts by removing any noisy portion of a trial that exceeded a threshold level of 1,000 μV (signal set to zero), which was chosen because extracellular neural activity did not exceed this threshold in a typical recording. If a trial had an artifact of > ~ 50 ms, it was eliminated from the analysis.

#### 2.5.4 EMG and displacement predictions via linear regression

After the elimination of trials with noise artifacts, multiple linear regression was applied to the data from each session for each recording array separately according to the following steps. Trials of each session were divided into 80% training and 20% test sets for cross-validation of the beta coefficients (every 5th trial was assigned as a test trial).

The following steps were followed for the training set.

##### 2.5.4.1 Splitting signals into different frequency bands

**Figure 4:**
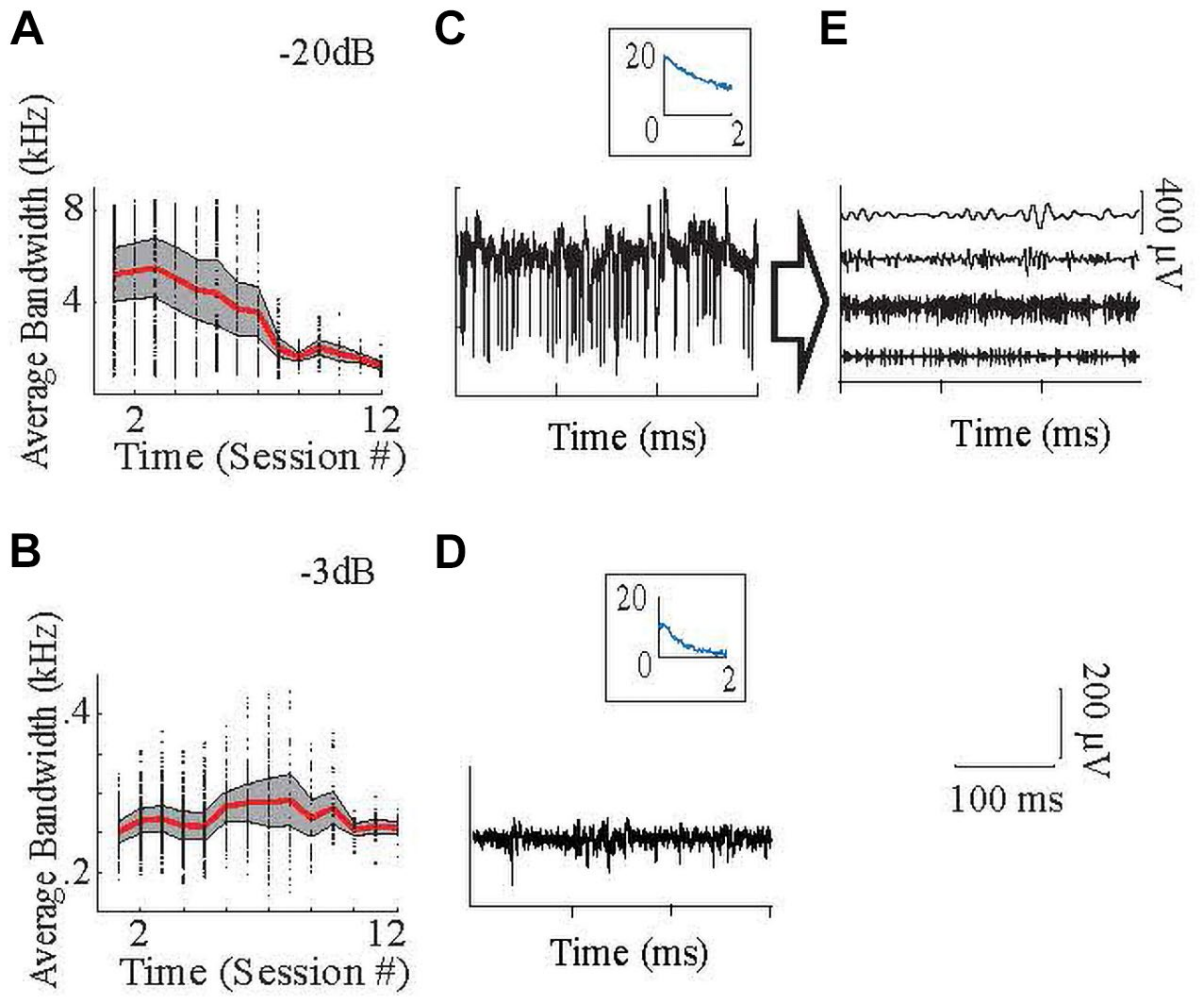
Frequency content of the signals. **(A–B)**: High end of the neural signal spectra, where the signal power declined to −20 dB **(A)** and − 3 dB **(B)** of the maximum as a function of frequency, averaged from all recording channels in all animals. Panel **(A)** with −20 dB amplitude cut-off, which shows that the high frequency components with small amplitudes are lost toward the end of the implant times. Signal components at such small amplitudes can still be utilized by the regression algorithm. Red is average, shaded area is +/− 0.5 SD. Dots are the data points for individual recording channels. Inset is the power spectrum [x-axis (kHz); y-axis (dB)]. Panel **(B)** with −3 dB amplitude cut-off, which shows that low-frequency signals (mainly LFP) were maintained throughout the study. **(C)** Raw sample neural recording with high spike activity, **(D)** Raw sample neural recording with lower frequency components, mostly in the LFP band. **(E)** Same recording in **(C)** after filtering with different bandpass filters to show that each frequency band contains different temporal patterns that are utilized by the regression (From top to bottom with the order of 30–100 Hz; 100–300 Hz; 300–1,000 Hz; 1,000–2,000 Hz.). The time calibration bar applies to **(C–E)**. The amplitude calibration bar applies to **(C,D)**.

Neural and EMG data were filtered with a 60 Hz offline band-stop notch filter. Next, EMG signals were band-pass filtered between 20–2,000 Hz using 4th-order Butterworth filters. Then, neural signals were divided into sub-bands by filtering with one of four band-pass filters (30–100 Hz, 100–300 Hz, 300–1,000 Hz, and 1,000–2,000 Hz) using 4th-order Butterworth filters to analyze separately the relationship of LFPs (lower two frequency ranges) and MUAs (higher two frequency ranges) with the EMG signal. Two example neural signals (Figures 4C,D) and one’s components (Figure 4E) after filtering in these frequency bands are seen in Figure 4. Thus, from each electrode array, 64 neural envelopes were generated (16 differential channels x 4 bands). The definitions of the LFP and MUA band frequency ranges vary greatly in the literature. The limits of LFP and MUA are generally defined between 0.5–500 Hz (Burns et al., 2010; Dubey and Ray, 2019; Lévesque et al., 2020; Baumel et al., 2021) and 300–10,000 Hz (Heck et al., 2007; Burns et al., 2010; Asan and Sahin, 2019; Dubey and Ray, 2019) respectively. Thus, the choices of the exact division points of these bands are somewhat arbitrary. To test whether our analyses were strongly dependent on our exact choices, some analyses were rerun after varying these points slightly (see sections 2.5.6 and 3.4).

##### 2.5.4.2 Generating EMG and neural envelopes

Following all the steps above, the trials in a session were concatenated to form continuous time signals. Next, filtered neural and EMG signals were full-wave rectified and low-pass filtered at 5 Hz to generate their signal envelopes. Transient filter responses at the start and end of the signals (‘edge effect’) were removed by truncating 0.1 s extensions of the data at each end, and then they were re-concatenated. Finally, the neural and EMG envelopes were down-sampled from 30 kHz to 1 kHz to reduce computer processing time.

##### 2.5.4.3 Regression

Beta coefficients of each of the 64 neural channels were calculated using Matlab’s built-in *regress* function (uses multiple linear regression model) based on EMG envelopes. The function uses the equation:


(1) $$y_{\mathrm{predicted}}=\sum_{i=1}^{64}\beta_{i}*x_{i}$$

where the dependent variable, y, is the EMG signal; the x_i_ (i = 1–64) are the neural signals; β_i_ are the respective regression coefficients.

To test the resulting regression model, the test set’s neural envelopes were generated as described for the training set and were run through the model to calculate the predicted EMG signals using the regression coefficients obtained from the training set. Finally, Pearson’s correlation (r) between the predicted and measured EMG signals of the test set was calculated for each session as a measure of the success of the prediction. To test the significance of the correlations, a random signal dataset was generated by replacing both EMG and neural data with an offline-generated noise signal of the same size, processing and filtering the noise signals the same as was done with the neural activity, obtaining the envelopes for these signals, performing the regression analysis, and calculating the correlation values between the predicted and actual EMG signals (‘chance level’). The number of random data sets generated equaled the number of actual data sets (n = 47,925).

##### 2.5.4.4 EMG and force outlier elimination

The data were tested in three different scenarios: (1) EMG outlier elimination, (2) force outlier elimination, and (3) no outlier elimination (all trials are kept). Outlier elimination aimed to ensure the repeatability of the behavior and compare these different methods in terms of how outliers affect the results. In each dataset, ±2 SD amplitude ranges were computed at each time point of the EMG and force envelope signals. Outliers were defined as any data point that fell above or below 2 SD of the EMG and force signal at any time point during the trial. Once a trial is tagged as an outlier, all the data collected in that trial, i.e., neural, EMG, and force, were excluded from the regression of the respective scenario.

###### 2.5.4.4.1 Displacement prediction

Using the same algorithm, displacements in the x- and y-axes, which were generated from the side view video recordings (Figure 3A), were also predicted from the synchronously recorded neural data of the 32-channel array. The neural data was processed the same way as in EMG prediction (section 2.5.4) and the displacement trajectories were processed the way it was explained above (section 2.5.2). The displacement data and the neural data were synchronized using force data as a reference. The time point at which force data exceeding 10% of maximum amplitude was synchronized to the frame in which paw passing the position of 3 mm behind the force bar, representing the first contact of the digits with the force bar. The behavior window was determined using displacement traces, the beginning being the frame in which lifting from the cage floor starts and the ending being the frame in which paw going back into the window and reaching the elbow level again.

#### 2.5.5 Regression for different phases of the behavior

The multiple linear regression algorithm was applied in each of the four time windows separately and to the movement as a whole (i.e., the entire behavior window as defined in section 2.5.3 and termed ‘All’) to investigate how the representation of the behavior in the neural signals varied across the movement phases. Additionally, the lifting, extension, and grasping phases were analyzed as a combined single phase (called ‘reaching’).

#### 2.5.6 Regression and mutual information analysis with time shifts

##### 2.5.6.1 Regression with time shifts

A shifted multiple linear regression algorithm was used to investigate how well neural signals predicted EMG signals at various latencies. First, the raw EMG data was shifted for different time lengths (±25, ±50, ±100, and ± 400 ms) with respect to the neural signals. Then, the multiple linear regression described above (section 2.5.4) was repeated for each shift separately to determine if the cerebellar signals encode information about the upcoming or preceding EMG activity. To shift in the negative direction in time (pre-movement), the amount of time equal to the shift was cut from the end of each raw, 4-s long neural signal, and the beginning of raw, same length EMG signal. Then their beginnings were aligned. Vice versa for positive (post-movement) shift.

##### 2.5.6.2 Mutual information with time shifts

Mutual information (MI) between the measured and the predicted EMG envelopes was calculated as a second measure of encoding of EMG by cerebellar activity. First, the envelopes of the signals were prepared in the same manner as described for the linear regression analysis. Next, probability distributions were computed from the envelope data sampled at 1 kHz to find the entropies. Then, assuming X and Y are two independent variables (in our case, neural and EMG envelopes), H(X) and H(Y) are their entropies, and H(X, Y) is the joint entropy between two variables, the MI between X and Y can be calculated as in (Cover and Thomas, 2005; Srinivasa, 2008):


(2) $$MI\left(X;Y\right)=H\left(X\right)+H\left(Y\right)-H\left(X,Y\right)$$

The calculation of these parameters is briefly as follows: The amplitude of the data is normalized to range between zero to one. The optimum bin size is determined based on the size of the data and the data is converted into bins of histograms. Then we compared bin data of neural and EMG signals and generated their probability matrix, P_xy_, which is composed of the probabilities of occurrences of every combination. Following, marginal probabilities of neural and EMG (P_x_ and P_y_) are found by summing the probability values in this matrix. Lastly, the final parameters H(X), H(Y), and H(X, Y) are found by calculating the summations of P_x_, P_y_, and P_xy, respectively,_ (Grinsted, 2024). For each of the 64 neural data (16 differential channels * 4 frequency bands), an MI value is calculated. Zero MI value indicates, no mutual information, while the difference from zero indicates the existence of mutual information. For the shift analysis, we calculated the MIs of every channel with varying shifts. For every shift, MIs averaged across channels, sessions, and arrays.

#### 2.5.7 Contributions of different frequency bands

To find the contribution of a particular neural frequency band to the overall prediction of EMG signal, beta coefficients from the training set belonging specifically to that frequency band were multiplied by the corresponding neural data for that frequency band from the test set (Eqs 3, 4. Similarly, for *y_100-300 Hz_, y_300-1,000 Hz_,* and *y_1,000–2,000 Hz_*, the *i* values range between 17–32, 18–48, and 49–64 respectively). Then, the explained variance values of the resulting time signals were calculated for each frequency band. Finally, its relative contribution was calculated as a percentage of the total variance. For example, for the 30–100 Hz frequency band, the contribution was calculated as in Eq. 5.


(3) $$\begin{array}{l} y_{\mathrm{predicted}}=y_{30-100\mathrm{Hz}}+y_{100-300\mathrm{Hz}}+\;y_{300-1,000\mathrm{Hz}}\\ +y_{1,000-2,000\mathrm{Hz}} \end{array}$$

(4) $$y_{30-100\mathrm{Hz}}=\sum_{i=1}^{16}\beta_{i}*x_{i}$$

(5) $$\begin{array}{l} \%\mathrm{Freq}.\mathrm{Band}\;\mathrm{Cont}._{30-100\mathrm{Hz}}=(\mathrm{variance}\left(y_{30-100\;\mathrm{Hz}}\right)\\ /\mathrm{variance}\left(y_{\mathrm{predicted}}\right))*\;100 \end{array}$$

A similar calculation was performed on the MI as an alternative measure. In this case, the sum of the MIs over all trials for the specific frequency band was divided by that of the sum for all frequencies and multiplied by a hundred.


(6) $$\begin{array}{l} MI_{\mathrm{Total}}=MI_{30-100\mathrm{Hz}}+MI_{100-300\mathrm{Hz}}+MI_{300-1,000\mathrm{Hz}}\\ +MI_{1,000-2,000\mathrm{Hz}} \end{array}$$

(7) $$MI_{30-100\mathrm{Hz}}=\sum_{i=1}^{16}MI_{i}$$

(8) $$\begin{array}{l} \%\mathrm{Freq}.\mathrm{Band}\;\mathrm{Cont}._{30-100\mathrm{Hz}}=(\mathrm{sum}\left(MI_{30-100\mathrm{Hz}}\right)\\ /\mathrm{sum}\left(MI_{\mathrm{Total}}\right))*100 \end{array}$$

#### 2.5.8 Clustering neural, and EMG data

To investigate the possibility of sub-patterns in the neural signals that were better correlated with the EMG signals, a k-means analysis was used. First, 1.5 s (1 s before and 0.5 s after the force maximum) long recordings were collected from all arrays and sessions (n = 2,477 for each EMG, and n = 12,992 for neural signals). 1 s before force maximum was divided into three equal (0.33 s long) time windows as lifting, extension, and grasping phases (The reasoning behind this approximation approach was that the clustering algorithm requires equal lengths of signals). 0.5 s after the force maximum was marked as the releasing phase. Following filtering (30–2,000 Hz 4th-order Butterworth band-pass filter), their envelopes were generated as in section 2.5.4. Then, envelopes were normalized to ±1 SD and zero mean using Matlab’s built-in *normalize* function (This function converts envelopes to z-score by extracting the average and dividing them to SD). Later, neural, and EMG envelopes independently clustered into five (*k* = 5) clusters using the *k-means* algorithm in Matlab [Optimum cluster number is found to be two for all data classes (neural and four EMGs). It was calculated using Matlab’s *evalclusters* function, with Calinski-Harbasz criterion and testing the data for between 1–10 clusters. Increased cluster number of *k* = 5 is used in order not to miss the smaller clusters in the data]. Finally, within each cluster *z*-score signals were averaged. The criteria to interpret the results was the timing of the increased amplitude or peaks of the *z*-scores (called ‘surge’) with respect to the phases of the reach-to-pull movement.

#### 2.5.9 Statistical methods

Most statistical comparisons were made using paired Student’s *t*-tests. One-way ANOVA and pairwise comparison with Bonferroni correction were used when appropriate. Other statistical tests are noted in the text when used. Means are given with their standard errors in the text and figures.

## 3 Results

### 3.1 EMG prediction

#### 3.1.1 Database

A total of ten CFMEAs were implanted in eight animals, and a total of 82 recording sessions were made in which the activity from one of the arrays was recorded (Table 1). Animals with two arrays generally performed two sessions on the same recording day, one for each array. A total of 5,595 trials were recorded. Of these, 400 trials were eliminated because of visually observable saturation or 60 Hz noise in the EMG signals, or incorrect reaching performance, as described in the Methods (section 2.5.2). In 291 trials, double reaching attempts were found, and each reach was analyzed as a separate trial. Thus, a total of 5,486 trials were analyzed (63 ± 29.7 trials per session), representing 93% of all trials recorded. When the recording channels were visually segregated into LFP recording channels and MUA recording channels, 71/160 channels were found to be recording MUA activity. These channels were recording high frequency MUA in ~1.5 sessions (n = 71 trials) on average out of ~8 sessions (mostly earlier sessions as seen in Figure 4), that makes MUA ~8% of the total database.

**Table 1:** Summary of the database used in this study.

	Database	
Label	Parameter	Count
A	Total Number of Implanted Animals	8
B	Total Number of Implanted Arrays	10
C	Total Session Count Across All Arrays and Animals	82
D	Total Recorded Trials Across All Sessions, Arrays, and Animals	5,595
E	Trials Eliminated Because of Noise	400
F	Double Reaching Attempts	291
G	Total Analyzed Trials	5,486
H	Average Trial Count Per Session	63 ± 29.7
I	Total Number of Force Outlier Trials	687
J	Total Number of EMG Outlier Trials Per Muscle (Wrist Extensor/Wrist Flexor/Biceps/Triceps)	1,199/1,224/1,279/1,214

Total analyzed trials (G) were calculated as D-E + F. Average trial count per session (H) is reported as mean ± standard deviation. Force (I) and EMG (J) outlier trials were searched for in all trials that were used for analyses (G), and were determined as described in the methods (see section 2.5.4).

##### 3.1.1.1 Prediction of reach-to-pull related EMG signals from cerebellar PML activity was demonstrated via cross validation

For each CFMEA, the 16 differential channels of neural recordings were divided into four frequency bands, and these 64 signals (16 × 4) were used in a regression model to predict the EMG signals, as described in the methods. The goodness of the model was then tested by correlating the predicted and actual EMG signals for each muscle for each session. The correlations between the predicted and actual EMGs were significantly higher than the chance level. Using all 5,486 trials, out of 328 linear regressions performed (82 sessions for four EMGs), 320 in the training sets (97.6%) and 263 in the test sets (80.2%) showed a statistically significant (*p* < 0.05, *t*-test for *r* ≠ 0) positive correlation (training set, *r* = 0.62.7 ± 0.19; test set *r* = 0.36 ± 0.22).

##### 3.1.1.2 Eliminating force or EMG outliers improve the results and ensure repeatability of the movement

Next, either the EMG or force outlier trials (see methods for outlier criteria) were eliminated from regression to investigate how they affect the correlation results. EMG outlier events were detected in 21–23% of the trials (wrist extensor: 1,199/5,486 trials, wrist flexor: 1,224/5,486 trials, biceps: 1,279/5,486 trials, triceps: 1,214/5,486 trials). When EMG outlier elimination was used, out of 328 linear regressions performed, 328 (100%, *r* = 0.66 ± 0.18) in the training sets and 254 (77.5%, *r* = 0.34 ± 0.23) in the test sets showed a significant positive correlation between the predicted and actual EMGs (*p* < 0.05, *t-*test for *r* ≠ 0). Force outliers were found in 687 (12.4%) trials. When force outlier elimination was used, out of 328 linear regressions performed, 324 (98.7%, *r* = 0.66 ± 0.20) in the training sets and 261 (80%, r = 0.38 ± 0.24) in the test sets showed a significant positive correlation between the predicted and actual EMGs (*p* < 0.05, *t*-test for *r* ≠ 0). Thus, different outlier elimination methods gave similar results, and there was a large overlap between the set of trials eliminated by different methods. However, the small difference of correlations in the test sets between force-outlier elimination and EMG-outlier elimination was nearly significant (*p* = 0.07) in favor of force-outlier elimination. Even though the difference between force-outlier elimination and no-outlier elimination was not significant (*p* = 0.34), we preferred to use the force-outlier elimination method to maintain the repeatability of the movement. Thus, only the force-outlier elimination method was used for further analyses.

##### 3.1.1.3 Cerebellar activity predicts both elbow and wrist, flexor and extensor EMGs with similar success

**Figure 5:**
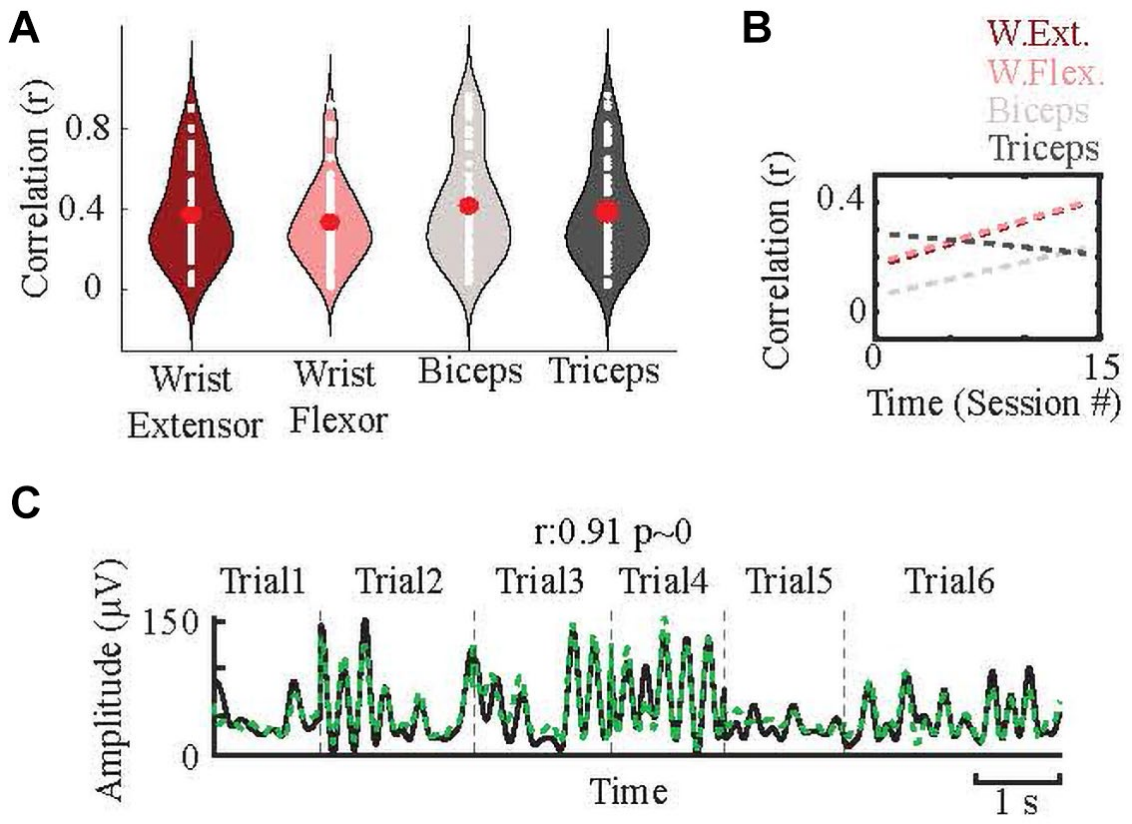
Prediction of forelimb EMGs. **(A)** Distributions (white dots) of the correlations between predicted and actual EMGs from all ten microelectrode arrays in all sessions (n = 82 for each EMG) and their averages (red dots). **(B)** The changes in the correlations (between predicted and actual EMGs) over time (sessions) for each muscle. Dashes are the line fits. Their slopes are: 0.017, 0.022, 0.008, and − 0.007 for wrist flexor, wrist extensor, biceps, and triceps, respectively. **(C)** Sample predictions for one EMG in a session with six trials in a test set, separated by vertical dash lines. Black: actual EMG envelope, green dash line: predicted EMG envelope.

The breakdown of sessions with a significant positive correlation (r > 0; *p* < 0.05) between neural and EMG signals were as follows: wrist extensor, 62/82; wrist flexor, 61/82; biceps, 65/82; triceps, 64/82. The average correlations in the test sets of these sessions were 0.37 ± 0.23 for wrist extensor, 0.34 ± 0.21 for wrist flexor, 0.42 ± 0.26 for biceps, and 0.39 ± 0.25 for triceps (Figure 5A). However, in the best cases, the correlations were much higher than these averages. For example, when the session with the best correlation from each of the ten CFMEAs were averaged for each EMG, r values were as followings: wrist extensor 0.53 ± 0.11; wrist flexor 0.85 ± 0.21; biceps 0.63 ± 0.20; triceps 0.52 ± 0.26. Figure 5C shows an example of the prediction results with high correlation. This suggests that PML signals are able to predict the EMG activity quite well in ideal conditions; however, as shown by the wide distributions in Figure 5A, the success rates varied considerably between arrays and across sessions. To understand the underlying factors of this variation, we looked into several possibilities.

##### 3.1.1.4 Prediction success improves over time

When the correlations from all the arrays were averaged to see the overall trend in prediction success over time, the fitted line showed a positive (0.008–0.022) slope for three out of the four EMGs (Figure 5B). When analyzing the arrays individually, 8/10 showed positive correlations, and three were significant (*p* < 0.05). High correlation values (>0.9) occurred mainly in the second half of the study period.

##### 3.1.1.5 Implantation area: higher correlations for arrays implanted closer to the paravermal vein

**Figure 6:**
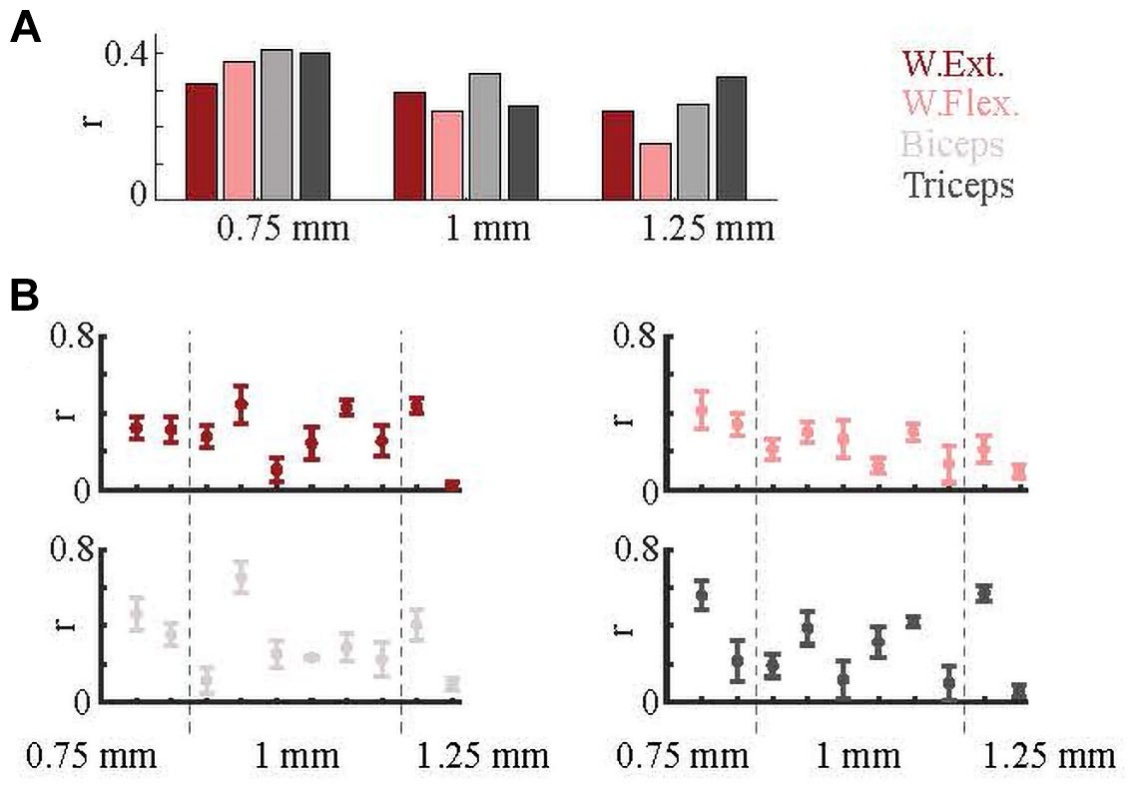
Effect of implant position. **(A)** Prediction results based on array implantation positions (0.75 mm, 1 mm, and 1.25 mm from paravermal vein). **(B)** Prediction correlations for individual arrays. Each of ten plots indicate one array. Means (dots) and standard errors (whiskers) are shown. Dash lines separate array groups based on their implantation positions.

Arrays were implanted in the PML such that the medial edge of the array was located at one of three mediolateral distances from the paravermal vein: ~0.75, 1.0, and 1.25 mm. Results were grouped according to the implant location to assess the dependence of correlations on the mediolateral position. On average, arrays at 0.75 mm predicted all EMGs with higher correlations (Figure 6A). We next investigated whether the relative success in predicting the EMG activity for different muscles varied across arrays. The success rates were similar for the wrist muscles across most of the arrays. Correlations with elbow muscles varied most across the arrays (Figure 6B). Based on multi-way ANOVA test (comparison between r-values vs. array location), the position on the PML was a significant factor (*p* = 0.04).

##### 3.1.1.6 The speed and the target end position variability may have decreased the prediction success in displacement prediction

Trajectories in x- and y-axes that were generated from the video images were tested for their prediction from neural data. The investigation on these kinematic parameters of reaching behavior can give deeper insight into the reaching task representation in the PML activity and the variability in the results that was shown above.

The correlations between predicted and measured displacement traces were all statistically significant (*n* = 20 predictions. Each prediction used *n* = 54 trials from a session of the same animal, for each dimension. Same session was analyzed with 20 different randomized selections of test set for both x- and y-axes. *p* < 0.05 was for all predictions). The predictions of x-axis traces were nearly significantly more successful than y-axis [The average r values for test sets were 0.25 versus 0.21 (*p* = 0.08) while for the training sets 0.51 versus 0.45, respectively, (*p* < 10^−15^)]. The prediction traces showed that the model predicted faster changes (Blue plots in Figures 3D,E) while measured traces (Red plots in Figure 3E) basically had one major event, the onset. To understand the results better, the predicted traces were low-pass filtered with a 2 Hz cut-off frequency (Black plots in Figures 3D,E), which improved the results (x-trace prediction success, average r = 0.31 and y-trace prediction success, average r = 0.27). Regardless, in any case x-axis was predicted with higher success.

To investigate the underlying reasons for better predictions in the x-axis, we calculated the average instant speed (Purple plots in Figure 3D). The average speed in the x-axis was approximately twice as fast as the y-axis (64 mm/s versus 31 mm/s). Another difference was the clear variability in the y-axis target end position, than the end position in the x-axis (Figure 3C, blue arrows), which was expected due to the force bar being a vertical stick and allowing multiple points for grabbing.

To this point, the results show that: (1) there is a significant correlation and prediction power between the forelimb EMG signals and the PML neural population activity; (2) eliminating force or EMG outliers improves the prediction; (3) small differences, in the order of 0.25 mm, in implantation site can affect the results; (4) forelimb kinematics can be predicted from neural data while speed and target end position may affect the results.

### 3.2 EMG predictions in different phases of forelimb reaching

For the analyses in this section and the following sections (3.3–3.5), the dataset was limited to the highest-correlated four sessions of each of the ten arrays, so that we could analyze the relationship between cerebellar and EMG activity in detail using datasets in which a relationship was clearly present. We next investigated whether the neural signals were better correlated with the EMG activity of specific phases of the reaching movement by performing the linear regression analysis on each of the four phases individually [lifting (L), extension (E), grasping (G), and releasing (R) and on the combined lifting, extension, and grasping phases (‘reaching’ phase, RC) and combined all four phases, ‘All’ (A)].

**Figure 7:**
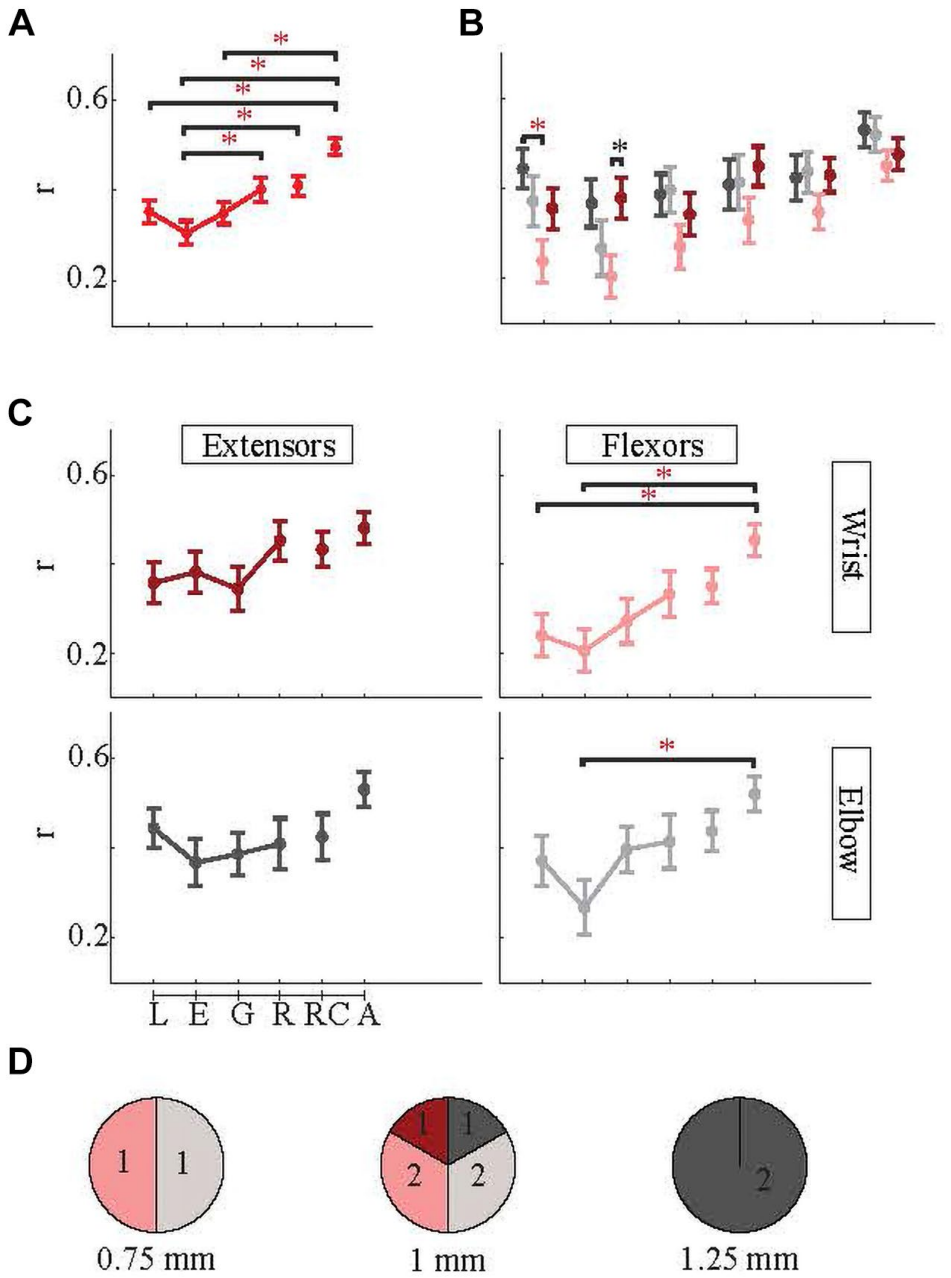
Correlations between predicted and actual EMG envelopes in individual phases of the behavior. Linear lines connect the consecutive phases. Dots are indicating the means, and the whisker plots are standard errors. Note: red asterisk, *p* < 0.05, and black asterisk *p* < 0.1. **(A)** Correlations for all four EMGs are averaged (red plots). **(B)** Correlations for individual EMGs in each behavioral phase. **(C)** Same as **(B)**, only arranged as flexor/extensor and wrist/elbow EMGs. **(D)** Based on implantation area (0.75 mm, 1 mm, and 1.25 mm from the paravermal vein) showing which EMGs were predicted with the highest success (r). Numbers indicate the number of arrays. Color codes for **(B,D)** are the same as in **(C)**.

The six distinct findings of this section were as follows. First of all, merging all phases (A) produced the most robust EMG predictions [*r* = 0.49 ± 0.23 for A in Figure 7A. A was significantly higher than L, E, and G phases (*p* < 3 × 10^−4^ for all with Bonferroni correction)] in individual muscles as it was when they were grouped, and A was similar for all muscles (Figure 7B) [For wrist flexor (light pink in Figure 7C), A was significantly higher than both L and E (Bonferroni correction *p* = 0.018 and *p* = 0.0029), and for the biceps (elbow flexors, light gray in Figure 7C) as well, A was significantly higher than E (Bonferroni correction *p* = 0.0135)]. Secondly, Combining L, E, and G into RC also created a higher correlation on average than each of the four phases alone [For RC in Figure 7A, r = 0.41 ± 0.27. The RC phase was significantly higher than the E phase (multiple comparison, *p* = 0.02 with Bonferroni correction)]. RC also had higher correlations than the R phase alone for all muscles individually except the wrist extensor whose R phase prediction was on par with RC phase (Figure 7B).

Third, going into more details, generally among four reach-to-pull components (L, E, G, and R), the later parts of the movement (G and R while R being more obvious) tended to be predicted better than the early parts of the movement (L and E) (Figures 7A,B). Fourth, the success of the prediction varied more in very early parts of the behavior (L and E) among four EMGs (Figure 7B) [ANOVA between four muscles in L phase showed significant difference (*p* = 0.03), and in this phase, elbow extensor (or triceps, dark gray in the figure) was significantly better predicted than the wrist flexor (light pink) (Multiple comparisons with Bonferroni correction, p = 0.01). Meanwhile, in the E phase, the difference among the four muscles was nearly significant (ANOVA *p* = 0.06), specifically the difference between wrist extensor and wrist flexor (Multiple comparisons with Bonferroni correction *p* = 0.12)]. This is most striking for flexors, both wrist and elbow (Figure 7C).

Fifth, most of the time E phase was the most difficult to predict (in Figure 7C, for all muscles except wrist extensors, E phase is the minima) while R prediction was generally the best among the four phases (in Figure 7C, for all muscles except elbow extensors, R phase is the maxima) followed by lifting phase most of the time. The L and G phases had intermediate correlation values (0.35 ± 0.31 and 0.35 ± 0.30 respectively). Sixth, wrist flexor predictions were mostly the least successful (light pink in Figure 7B) while elbow muscles was generally the most successful.

Arrays were also grouped according to which muscle EMG that they best predicted (Figure 7D). The distribution varied with location. At 0.75 mm, the arrays best encoded the EMG activity of the flexors (biceps or wrist flexor). Arrays at 1 mm represented all four EMGs with similar frequency. At 1.25 mm, both arrays were a better predictor of the triceps EMG.

#### 3.2.1 Video-based sessions

Lifting and the extension phases were re-analyzed based on their trial-specific, precise lifting and extension phase time marks. Average lifting and extension phase lengths were respectively, 0.283 ± 0.262 and 0.444 ± 0.273 ms (n = 154 trials). Most of them showed improved prediction success (15/24 increased correlation), but the difference was not significant (*p* = 0.6, t-test).

### 3.3 Regression and mutual information with time shifts

This analysis aimed to get insight into the sensorimotor content of the recorded neural signals. If correlations between predicted and measured EMGs increased when the measured EMG signals were shifted in the negative time direction (i.e., neural activity predicted future EMG activity), it would be evidence that the neural activity contained pre-movement-related information, and in contrast, if correlations increased with positive EMG shifts, it would provide evidence for the neural signals encoding post-movement information, perhaps sensory feedback caused by the movement (Cerminara et al., 2015). In this analysis, the entire behavior window (from the beginning of the lifting phase to the ending of the releasing phase) is used.

#### 3.3.1 PML signals carry both pre-movement and post-movement information

**Figure 8:**
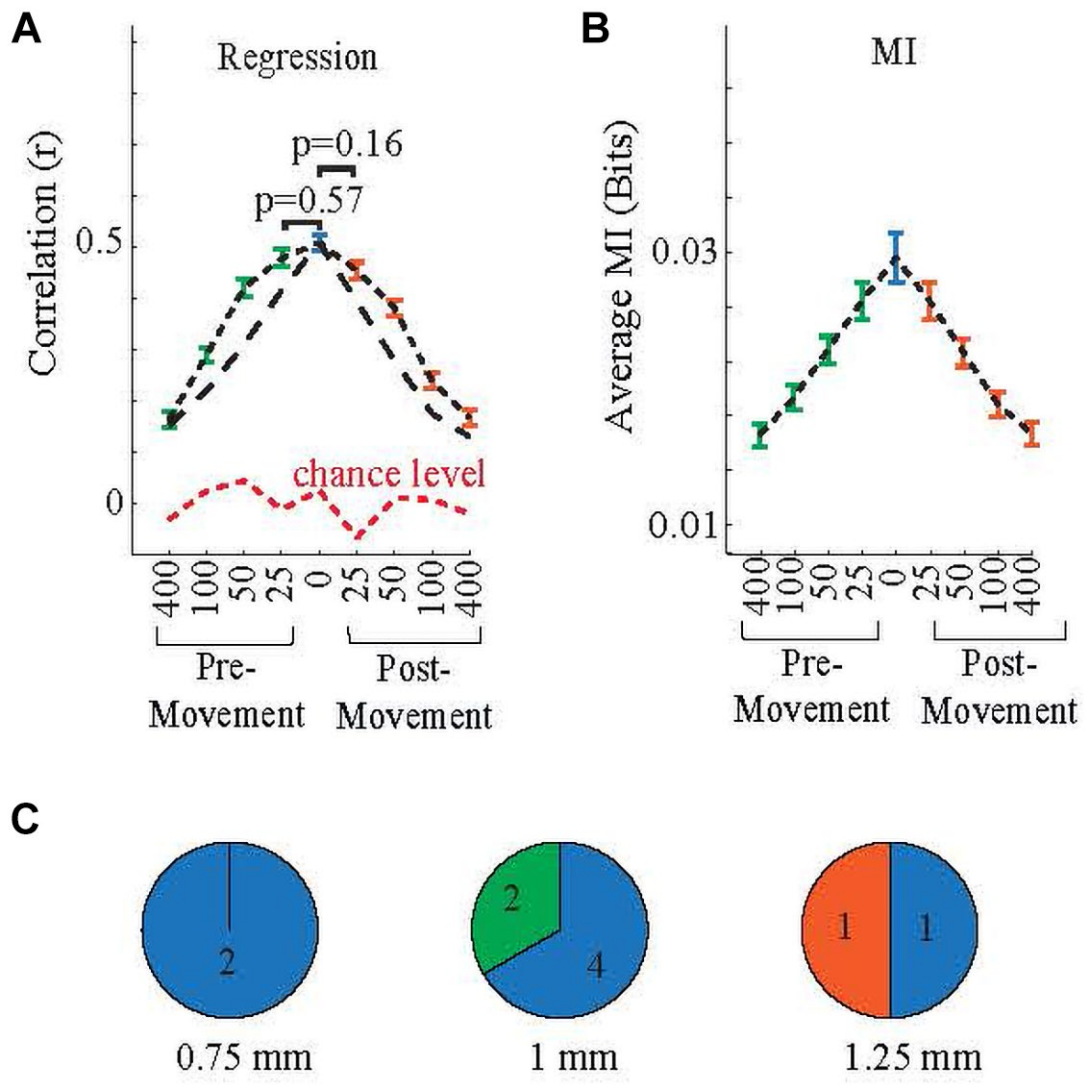
Group correlations from linear regression **(A)** and mutual information **(B)** for forward and backward shifting of neural data in time with respect to the EMG signals to determine the post-movement and pre-movement content of the neural signal. Whisker plots show the standard error. (Using 5 Hz filter: black short dashes, 10 Hz filter: black long dashes), while green and orange whisker plots are, respectively, pre-movement and post-movement. The blue bars are for ‘no shift’ (or ‘synchronized’), while green and orange bars, respectively, pre-movement and post-movement. *p* values between ±25 ms and no-shift cases do not show significance in both panels. The red dash line in **(A)** indicates the chance level, i.e., predictions using random neural signals. **(C)** Arrays are grouped based on their highest correlated shift based on regression as ‘pre-movement’, ‘post-movement’, or ‘synchronized’. Numbers indicate the number of arrays. Color codes are same as in **(A,B)**.

The correlations are shown in Figure 8A for various time shifts in both directions. Except for ±25 ms shifts, all other time shifts had significantly lower correlations than the no-shift case (multiple comparison test: pre-movement25 vs. no-shift *p* = 0.57; post-movement25 vs. no-shift *p* = 0.16, other shifts vs. no-shift *p* < 0.05). However, the fact that a significant correlation exists for delays as long as 400 ms in either direction [The difference between any and every shift and chance level is significant (p < 0.05)] suggests that the PML signals contain both post-movement- and pre-movement-related information (Figure 8A, short black dashes). The regression analysis was repeated with lighter filtering (10 Hz corner frequency instead of 5 Hz) on the rectified neural and EMG signals for finding the envelopes (Figure 8A, long black dash line) to test whether the correlations were artifacts due to low corner frequency of the filter, because the time constants (1/2πf) for first-order 5 Hz and 10 Hz filters are 31.8 ms and 15.9 ms, respectively. The results for correlations with 5 Hz and 10 Hz filters were similar, except that correlations were smaller for all shifts, particularly the ±50 ms shift, with the 10 Hz filter (Figure 8A). This suggests that the 5 Hz envelope filter had a negligible impact on the regression results, i.e., did not cause false correlations for non-zero time shifts. Mutual Information plots (Figure 8B) agree with the correlation results (all shifts vs. no-shift p < 0.05, except pre-movement and post-movement 25 ms). Thus, the neural data contained both pre-movement and post-movement information that decreased with increasing time shifts. For both methods, the correlations were above the chance level at all time shifts (*p* < 10^−9^). In addition, both methods overall showed slightly higher values for negative (pre-movement) time shifts relative to their corresponding post-movement shifts, suggesting a higher level of pre-movement information content in the neural signals; however, the differences did not reach statistical significance.

#### 3.3.2 Sensorimotor information and the representation of EMGs depend on array location

Even though on average the arrays showed the highest correlations for the no time shift case, this was not the case for some arrays. Arrays were categorized as ‘post-movement’, or ‘pre-movement’ or ‘synchronized’, depending on which shift produced the best EMG prediction (positive, negative, or zero-time shift, respectively). Out of ten arrays, one was post-movement, two were pre-movement, and seven were ‘synchronized’. This classification appeared to be related to the array’s position on the PML (Figure 8C). Arrays classified as ‘synchronized’ were found at all three implantation distances from the paravermal vein. However, both pre-movement arrays were located at 1 mm, and the only post-movement array was implanted at 1.25 mm.

### 3.4 Frequency band contributions

#### 3.4.1 High frequencies contribute more to the EMG prediction

**Figure 9:**
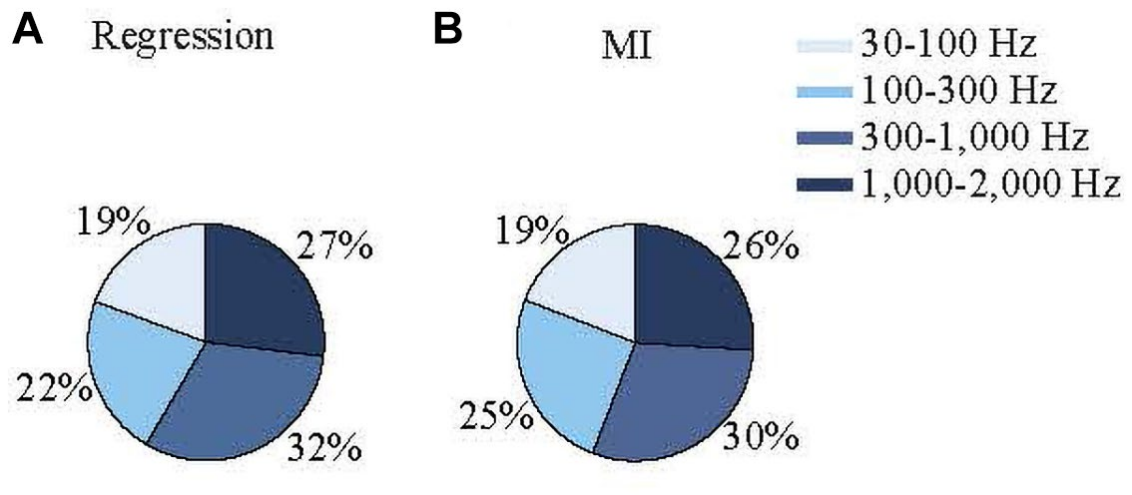
Percent contributions of neural signals in various frequency bands to the predicted EMG envelopes based on regression **(A)** and mutual information **(B)** using signal variances (See Eqs 2, 6–8).

Different frequency bands in extracellular electrophysiological recordings are thought to reflect different aspects of neuronal signal processing (e.g., LFPs and spike activity). Therefore, determining the relative contributions of each frequency band to the EMG prediction could shed light on which components of the cerebellar activity contribute to the EMG activity. For both the regression and MI models, neural activity was filtered into four frequency bands: 30–100 Hz, 100–300 Hz, 300–1,000 Hz, and 1,000–2,000 Hz. The contribution of each band to the total variance can be determined by forcing the coefficients for the other frequencies zero in the regression model and rerunning the predictions (Eqs. 3–7, see methods). Of the four bands tested, the 300–1,000 Hz band made the largest contribution (32 ± 19%) and the 30–100 Hz band contributed the least (19 ± 18%) to the EMG predictions based on the signal variances of each band (Figure 9A). The difference between all frequency bands was significant (ANOVA, *p* = 5 ×10^−9^). The differences between 30–100 Hz and 300–1,000 Hz bands; 30–100 Hz and 1,000–2,000 Hz bands; and 100–300 Hz and 1,000–2,000 Hz were significant (multiple comparisons, *p* < 0.0011 for all).

To test whether these differences were sensitive to the specific limits of the bands, we reran the analysis after shifting the limits to slightly higher (30–120; 120–350; 350–1,100; 1,100–2,200 Hz) and lower (30–80, 80–250; 250–900; 900–1,800 Hz) frequencies. The changes in the results were not significant (p ranged between 0.1–0.92 for t-tests). When they were simply divided into low- and high-frequency bands (30–300 Hz and 300–2,000 Hz), their percentage contributions were 42.7 ± 20% and 57.3 ± 20%, respectively, (*p* = 1.5×10^−20^).

The percent contributions of each frequency band from the MI analysis were consistent with the regression results (Figure 9B). The 300–1,000 Hz band showed the highest MI between the neural and EMG envelopes (29 ± 5%). The difference between all frequency bands was significant (one-way ANOVA, *p* = 1.6×10^−48^), except between the 100–300 Hz and 1,000–2,000 Hz bands. These results show that overall high-frequency components contribute to the prediction of EMG signals more than the lower frequencies.

### 3.5 EMG and neural signal peaks mark behavioral phases

**Figure 10:**
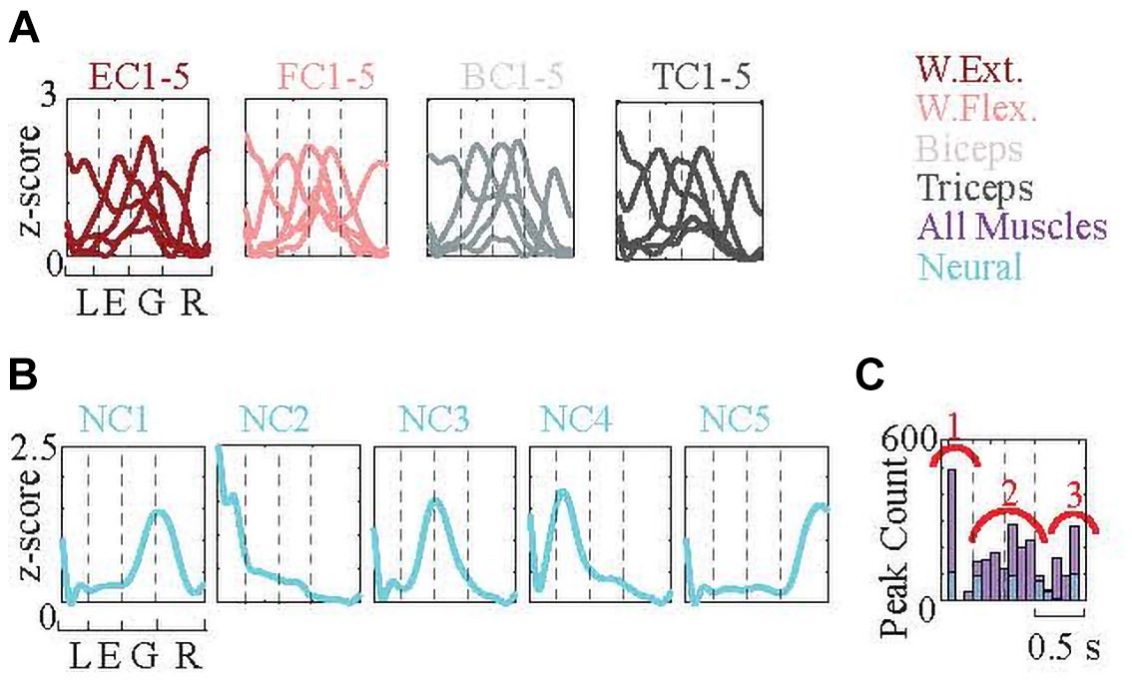
k-means clustering of normalized neural signal, EMG signal, and force profile envelopes. (The numbers of signals in each cluster: wrist extensor 263, 539, 535, 526, 406; wrist flexor 443, 44, 587, 524, 271; biceps 522, 522, 206, 533, 486; triceps 297, 439, 489, 550, 494; neural 2,784, 2,298, 2,439, 2,457, 2,982). **(A)** Each signal type (four EMGS and neural data) is clustered into five clusters independent of others and the means of the clusters are plotted. Signals capture from 1 s before the force peak to 0.5 s after the peak. Behavioral phases (approximated) are delineated with the dash lines. Each cluster is encoding different parts of the behavior. **(B)** The histogram shows the distribution of cluster peaks over the behavior window. Clustering was repeated 100 times. One of them is displayed in (A). The cluster peaks mostly occur at the beginning, middle, and end. Neural cluster peaks are marked with blue color, which shows a homogenous distribution that initiates each one of the four phases and terminates the phase.

Each set of signal amplitudes was normalized (converted to z-scores as explained in Methods), and the signal envelopes from each set (neural and four EMGs) were clustered using a k-means algorithm into five independent clusters. This clustering was repeated 100 times to increase robustness. Examples of the five EMG cluster-means for each muscle are shown in Figure 10A (wrist extensor, EC1-5; wrist flexor FC1-5; biceps, BC1-5; TC, triceps1-5), and of the neural cluster (NC) means in Figure 10B (NC1-5). For all 100 cluster runs, the peak times of each of the 25 (five neural and 20 EMG clusters) cluster-means were used to generate a histogram of their distribution in the behavior window (Figure 10C). The histogram shows that the EMG profiles (purple bars) had, 1 - a strong peak at the lifting phase, 2 - a widespread distribution throughout the middle phases, and 3 - another distinct peak during the releasing phase. NC peaks (blue bars) occurred at the beginning of each one of the four phases and the ending of the last phase. In summary, clustering analysis showed that the cooperative work of neural and EMG signals starts, proceeds, and terminates the behavior (Figure 10B).

#### 3.5.1 EMG patterns correlate to elbow and wrist angles

**Figure 11:**
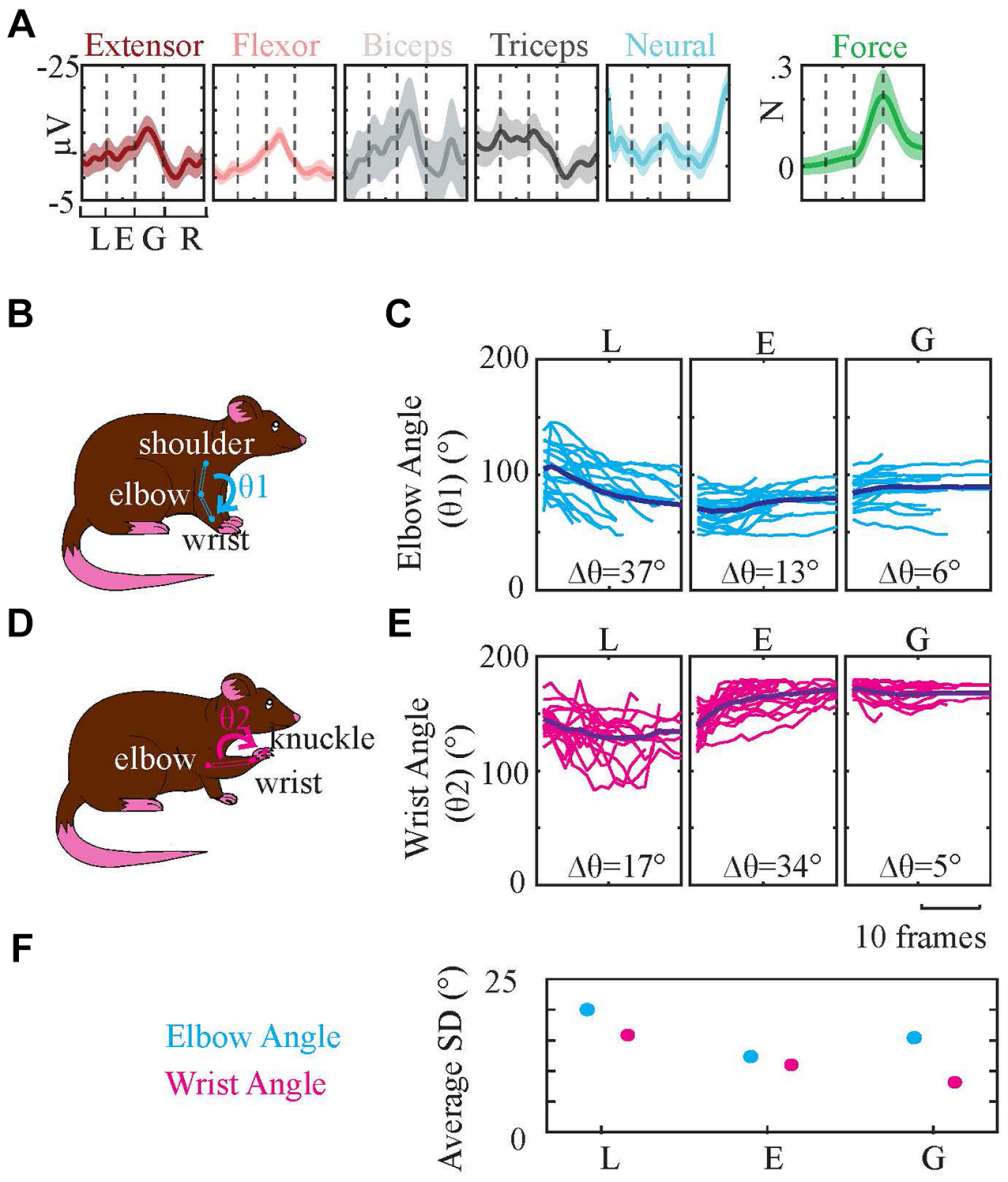
Averaged EMG Traces and Elbow and Wrist Angle Change During Behavior. Based on video recordings elbow and wrist angles of three reaching phases are measured. **(A)** Signals are averaged and their SDs are calculated (shaded areas). (Since the data is composed of all animals and sessions, SDs across them were large. For clarity of results, we used 5% SD for EMGs, 1% SD for neural data, and 50% SD for force data. And the neural data amplitude is multiplied by five.) **(B)** demonstrates elbow angle (θ1) measurement by tracing shoulder, elbow, and wrist. **(C)** shows the changes in θ1 across video frames measured at lifting, extension, and grasping phases. **(D)** demonstrates wrist angle (θ2) measurement by tracing elbow, wrist, and knuckle (between the second and third fingers). **(E)** shows the changes in θ2 across video frames measured at lifting, extension, and grasping phases. Δθ is the maximum angle change among all the traces in that plot. **(F)** Average SDs of wrist and elbow angles in **(C,E)**.

The signals grouped in the clustering section were further analyzed by averaging (without normalization) to determine their overall followed trajectories (Figure 11A) during reach-to-pull behavior. The elbow and wrist angles were also measured using video recordings to investigate the relationships between the recorded EMG trajectories in Figure 11A and joint angles (Figures 11B–E) to get a deeper understanding of kinematic parameters of the reach-to-pull behavior.

During lifting elbow angle changes the most [Figure 11C, Δθ (the maximum angle change) is 37 degrees] which coincides with increased triceps activity during the lifting phase in Figure 11A (the fourth plot). During the grasping stage, the wrist angle was quite stable (Figure 11E, Δθ is 5 degrees), which agrees with the finding in Figure 11A that shows increased activity of both wrist extensor (the first plot) and wrist flexor (the second plot) to stabilize the wrist angle. The wrist angle changed the most during the extension phase (Figure 11E, Δθ is 34 degrees) as a result of repositioning the wrist after lifting (Figure 11E, Δθ is 17 degrees) and preparing for grasping. There was still some elbow angle change during grasping even though it is the smallest in all. Both biceps (Figure 11A, the third plot) and triceps (Figure 11A, the fourth plot) EMG amplitudes are elevated during the lifting, extension, and grasping phases before a sudden dipping at the end of the grasping phase. These angle measurements in general agree with the EMG profiles.

Lastly, we tested the data for the variability of joint angles during three reaching phases, between different trials (Figure 11F). Results showed statistically higher SD in early phases (lifting phase specifically) compared to the last phase of the reaching, releasing phase, in general [Based on ANOVA test, the difference between SDs of lifting, extension, and releasing phase elbow angles was significant (*p* < 10^−24^). Specifically, the lifting phase SD was significantly larger than both extension and releasing phases SDs (p < 10^−14^). Likewise, ANOVA between SDs of lifting, extension, and releasing phase wrist angles showed a significant difference (p < 10^−15^), and the difference between lifting and both two other phases was significant (p < 10^−8^)]. These results also correlate with phase analysis results in section 3.2, which indicated an increased prediction success as phases progressed in the behavior window.

## 4 Discussion

In this study, we recorded the neural activity from the PML of the cerebellar cortex with novel multi-electrode arrays, with the rationale that a larger number of recording channels could yield more robust information that can shed light on the involvement of the cerebellum in a motor behavior. In particular, we aimed to record MUA and LFPs with these arrays rather than targeting single-cell spiking activity, to sample a larger population of neurons for making predictions about the ongoing motor act. Our results show the feasibility of obtaining recordings from chronically implanted arrays in cerebellar cortex and that MUA and field activity can be used to predict aspects of a complex goal-oriented motor behavior, reaching to a target.

### 4.1 EMG predictions

A major goal for this study was to investigate how well cerebellar-PML activity could predict EMG activity during reaching movements. In our best recordings, we indeed were able to obtain strong correlations between PML and EMG activity, and using a regression model, we could accurately predict the actual EMG patterns from the PML activity (e.g., Figure 5C).

The MUA (300–2,000 Hz) contributed the most to EMG predictions even though it had less power than the LFPs in the recorded signals. The fact that the contributions from the 30–300 Hz band are lower suggests that most of the information is contained in the spiking activity of the neurons rather than the LFPs that are typically associated with synaptic signals. Although smaller, the contributions from the LFP band were not insignificant, which could also be originating from the high-frequency cerebellar oscillations (150–300 Hz) from the cerebellar cortex (Cheron et al., 2004; Groth and Sahin, 2015).

The correlations between the predicted and the measured muscle signals suggest the presence of forelimb-related information in the PML cortex of the cerebellum. For some animals, the regression algorithm predicted signals with correlations as high as 0.8 on average over multiple sessions and days. However, the goodness of the prediction varied considerably across recordings. This variation has multiple sources, at least some of which we identified. 1- Array implantation location on the brain was a factor. In our experiments, activity in arrays closer to the paravermal vein had better prediction success. The possible involvement of the multizonal cerebellar activity in a given motor function (Aoki et al., 2019) is a challenge while sampling related information of a function. Our arrays were covering ~0.5 × 0.5 mm area on the cerebellar cortex, which were probably limited to one or two neighboring zones. 2- Implant age also affected the quality of prediction. The data showed that prediction results improved over time, which may be attributed to the healing of tissue damage after the first trauma caused by implantation. 3- The goodness of the predictions varied across muscles. Extensor muscles, specifically the triceps (Figure 7B), showed a better success rate for the reaching behavior tested in our study while the wrist flexor had the lowest prediction success among the four muscles. 4- Lastly, prediction success varied between movement phases. The later phases were generally better predicted for most muscles, whereas the success in predicting EMG activity during the earlier phases varied more. This can be due to the variability in the movement trajectories, particularly in the earlier phases, that was shown with displacement tracing (Figure 3C) and elbow/wrist angle measurement (Figure 11).

### 4.2 Variations in phases of forelimb reaching

In mice, reaching movements take about 0.5–0.8 s (Becker et al., 2020). In Gok’s study (Gok and Sahin, 2019), the behavior window was divided into two phases, ‘reaching’ (−0.2 s from the center of the behavior) and ‘grasping’ (+0.2 s). Here we considered the timepoint at which force amplitude exceeds 10% of the maximum force amplitude as the beginning of grasping and the ending of the reaching phase. In this study, we also investigated the earlier parts of the reaching, i.e., lifting and extension. The regression algorithm was applied in each one of the four time windows separately, and also as a whole. This analysis revealed that the algorithm can predict the lifting and releasing behaviors, in general, better than extension.

For the lifting and extension time windows, for most of the data, we used an average time window of 0.35 s based on a pilot investigation we did to estimate the length of these windows. In three sessions from three different animals, we used the video images to trial-specific marking of these time windows, and this approach improved the prediction success. This shows the variability in these behavioral window lengths and the fact that higher prediction success would be possible if all the data was analyzed in this manner. However, in the current study manually marking these windows was prohibitively time consuming. In a future study, using a movement tracking software to determine the precise movement phase transitions for every trial, more accurate results would likely be achieved.

It has been suggested that the cerebellum is involved chiefly in the initiation and termination phases of the movement (Scudder et al., 2002; Gaffield et al., 2011). Consistent with this, cerebellar activity is often best correlated with the duration of the movement [for review, see (Lang, 2015)]. The higher prediction correlations for the lifting (beginning stage) and releasing (ending) phases in our data support this idea in general. Specifically, we found a high level of regression success both at the initiation (lifting) and termination (releasing) phases for both wrist and elbow flexors and elbow extensor (triceps) muscles, although the wrist extensor showed increased prediction success in the extension phase.

### 4.3 Sensory vs. motor content of signals

For optimum motor control of voluntary functions, the cerebellum integrates sensory and motor information that it receives via its connections from the sensorimotor cortices and with the spinal cord. The cerebellum can handle complex movements like reaching with its internal models that are dynamically updated through forward or feedbacks loops to predict sensory inputs and motor commands in an adaptive manner (Kawato and Wolpert, 1998; Wolpert et al., 1998; Bell et al., 2008). In this study, shifting the neural signals in the time domain with respect to EMG signals in both pre-movement and post-movement directions resulted in lower, but still statistically significant, correlations (Figure 8A), as shown by regression, and substantiated by mutual information measure between the neural and EMG signals (Figure 8B). Testing with a 10 Hz corner frequency of the envelope filter revealed that the observed correlations with time shifts were not simply due to the smearing of data in time by the low-pass filter. It should be noted that the time shifts considered here are much longer than the propagation times that efferent or afferent neural signals would take to travel between the CNS and the muscles, which are typically ~10–15 ms in the rat. Therefore, the time-shifted correlations hint at the generation times of the signals in the cerebellar cortex with respect to the muscle activity that can occur with much larger delays or advances than their propagation times through the peripheral nervous system.

The results can be interpreted as PML carrying both sensory and motor information, agreeing with reports on cerebellar neuroanatomy suggesting that the cerebellum’s paravermal zones are involved in integrating sensory inputs with motor commands to coordinate execution of the motor function (Knierim, 2019) through its strong connections to M1 and S1 (Evarts and Thach, 1969; Aoki et al., 2019). Additionally, when the response to the shifts in positive and negative direction was analyzed for individual arrays, the results showed that peak correlations were generated at pre-movement shifts for some arrays. For others, it was at the post-movement shifts. This result, again, is in agreement with the idea that the cerebellar cortex is receiving both descending information from the motor cortex and ascending sensory information from the periphery. Nevertheless, in all cases in Figure 8A except the 400 ms shift, negative shifts (pre-movement) showed slightly higher correlations than the positive shifts.

However, how, why, or to what extent this coexistence of motor and sensory information in the cerebellum occurs is complicated to determine. For example, the cerebellum may control movement through acquiring the sensory information about the environment (Manto et al., 2012). Also, the sensory inputs are generally correlated with motor outputs (Paulin, 1993), therefore dissecting motor information in the cerebellum is very challenging, especially for the period following movement initiation, where it can be a mixture of both types of information.

### 4.4 Implant location on the cortex: execution vs. planning

Traditionally, PML paravermal zones are thought to be involved in correcting errors during movement execution while the lateral cerebellum was believed to plan and initiate the movement (Allen and Tsukahara, 1974). It is also known that the C zones in the PML, that correspond to paravermal zones, are associated with the forelimb reaching (Apps and Hawkes, 2009); they project to the IPN (Apps et al., 2018) and contribute to the spatial and temporal coordination characteristics during motor execution in trained forelimb reach movements (Milak et al., 1997).

Later studies showed that multiple cortical areas might have parallel projections to the cerebellum, and the functional distinction between the interpositus and lateral nuclei may be less definitive [for review, see (Lang, 2015)]. In our data, the lower prediction correlations at long time shifts (e.g., 400 ms pre-movement shift) relative to the no-shift correlations are in agreement with the idea that the intermediate areas (or paravermal zones) of the PML, where we performed most of our recordings as well, are primarily involved in the execution of the movement rather than its planning (Zhu et al., 2023). However, the investigation of even earlier stages (earlier than 400 ms) would address this issue more definitively, considering that the preparation phase of the reaching task may start earlier.

### 4.5 Clusters of patterns

All four EMG traces had at least one cluster with a local peak in activity in at least one of the four phases (Figure 10A). This implies that each one of the forelimb muscles may become active in different phases of the behavior and suggests a large variability in the recruitment of these four muscles in a restricted and relatively simple behavior in a highly trained animal. However, their collaborative effort starts, maintains, and terminates the behavior (Figure 10B). The neural signals also show various patterns, some mainly encoding the beginning of the behavior, some the middle, and some the end. Interestingly, all EMGs and neural data had some clusters with three bumps showing activity in multiple phases (Figure 10B). This agrees with the reports showing that PC activity had variable patterns during the locomotion (Sauerbrei et al., 2015).

## Data availability statement

The raw data supporting the conclusions of this article will be made available by the authors, without undue reservation.

## Ethics statement

The animal study was approved by the Institutional Animal Care and Use Committee (IACUC), Rutgers University, Newark, NJ. The study was conducted in accordance with the local legislation and institutional requirements.

## Author contributions

MS and EL contributed to conception and design of the study and edited the manuscript. EC and MS performed the experiments. EC collected and analyzed the data, and wrote the first draft of the manuscript. All authors contributed to manuscript revision, read, and approved the submitted version.
